# Polyelectrolytes Assembly: A Powerful Tool for Electrochemical Sensing Application

**DOI:** 10.3390/s20113211

**Published:** 2020-06-05

**Authors:** Ivana Škugor Rončević, Denis Krivić, Maša Buljac, Nives Vladislavić, Marijo Buzuk

**Affiliations:** 1 Department of General and Inorganic Chemistry, Faculty of Chemistry and Technology, University of Split, 21000 Split, Croatia; skugor@ktf-split.hr (I.Š.R.); nives@ktf-split.hr (N.V.); 2 Division of Biophysics, Gottfried Schatz Research Center, Medical University of Graz, 8036 Graz, Austria; denis.krivic@medunigraz.at; 3 Department of Environmental Chemistry, Faculty of Chemistry and Technology, University of Split, 21000 Split, Croatia; masa@ktf-split.hr

**Keywords:** polyelectrolytes, electrochemical, sensors, layer-by-layer, dip-coating, drop-casting, electroanalytical, biosensors, chemical sensors

## Abstract

The development of sensing coatings, as important sensor elements that integrate functionality, simplicity, chemical stability, and physical stability, has been shown to play a major role in electrochemical sensing system development trends. Simple and versatile assembling procedures and scalability make polyelectrolytes highly convenient for use in electrochemical sensing applications. Polyelectrolytes are mainly used in electrochemical sensor architectures for entrapping (incorporation, immobilization, etc.) various materials into sensing layers. These materials can often increase sensitivity, selectivity, and electronic communications with the electrode substrate, and they can mediate electron transfer between an analyte and transducer. Analytical performance can be significantly improved by the synergistic effect of materials (sensing material, transducer, and mediator) present in these composites. As most reported methods for the preparation of polyelectrolyte-based sensing layers are layer-by-layer and casting/coating methods, this review focuses on the use of the latter methods in the development of electrochemical sensors within the last decade. In contrast to many reviews related to electrochemical sensors that feature polyelectrolytes, this review is focused on architectures of sensing layers and the role of polyelectrolytes in the development of sensing systems. Additionally, the role of polyelectrolytes in the preparation and modification of various nanoparticles, nanoprobes, reporter probes, nanobeads, etc. that are used in electrochemical sensing systems is also reviewed.

## 1. Introduction

Polyelectrolytes (PEs) are macromolecules that combine polymeric and electrolyte properties, which allow them to be used in a variety of applications. The main property of PEs is that, when dissolved in a polar solvent, their functional groups undergo ionization. The solubility of a PE in certain solvents depends on its structure, functional groups, the presence of a counter ion, pH, etc. PEs are, in general, soluble in water. However, the ionization of some polyelectrolytes can be observed in polar organic solvents (e.g., dimethylformamide) [1] or a mixture of water and dimethylformamide [2]. Depending on the functional group charge present at the polymer backbone after ionization, PEs can be classified as cationic or anionic (e.g., homogenous polyelectrolytes) and ampholytic (if both positively and negatively charged functional groups are present). An ampholytic (sometimes termed as amphoteric) polyelectrolyte is a copolymer comprising more than one monomer attached to its backbone, with at least one of them containing a positively charged functional group and at least one of them containing a negatively charged functional group. Besides the presence of monomers with charged functional groups, copolymer polyelectrolytes can comprise monomers that feature no ionizable functional groups. The net charge of such copolymer polyelectrolytes is determined by the nature and number of their charged units. Similar to the classification of “common” electrolytes, polyelectrolytes can be classified as “strong” ones or “weak” ones, depending on the degree of ionization. The degree of ionization (or, more properly, the average degree of dissociation) is a property that depends mostly on the nature of chemical properties of ionizable functional groups. Accordingly, polyelectrolytes comprising a functional group that exhibits a constant degree of ionization over a wide pH range are referred to as “strong” polyelectrolytes. Those PEs whose functional group ionization degree is pH-dependent are classified as “weak” polyelectrolytes (there are characterized with a p*K* value—intrinsic dissociation constant). Additionally, the p*K* value can be used as criterion for the classification of the polyelectrolytes: those with p*K* values between 0 and 14 can be classified as “weak,” while those with p*K* values less than 0 and greater than 14 can be considered “strong” [3]. 

It follows from the above that one of the most important properties of the PEs is their ability to interact with species bearing a net electric charge opposite to their own. If the oppositely charged species is a monomeric acid or base, a polysalt product is obtained. 

In addition, the electrostatic interaction between two oppositely charged PEs results in the formation of a polyelectrolyte complex (PEC). PEC formation is affected by polyelectrolytes’ properties such as steric factors, the length and rigidity of the PE backbone, the nature and charge density of ionizable functional groups, and polyelectrolyte concentration (that affect the viscosity of solution, degree of ionization, etc.). Additionally, PEC formation can be tuned via the manipulation of solvent properties (pH, temperature, etc.) or composition (the presence of various salts, other polyelectrolytes, etc.) [4,5,6]. PECs can be present in a variety of morphologies, including films, fibers, capsules, nanoparticles, tapes, tubes, or even coacervates [7]. A closer look at phase behavior and driving forces responsible for PEC formation was presented by van der Gucht et al. [4]. Furthermore, the dependence of PEC formation and morphology upon parameters such as the nature of the polyelectrolyte, ionic strength, and pH was discussed. The authors showed that the electrostatic driving force is multi-responsive, as: (i) the strongest driving force is obtained at charge stoichiometry, (ii) discharging one of the components of PE micellar network by increasing pH leads to network disintegration, (iii) the addition of salt weakens driving force leading to a decrease of the aggregation number of micelles and consequently to the lower viscosity of a system. The latter is a consequence of the fact that upon increasing salt concentration, the entropy increase for the released counter ions becomes smaller. However, in the case when the driving force for PEC formation is very large (strong polyelectrolytes at low salt concentration), the thermodynamic equilibrium state may not easily be reached, and, therefore, the final structure is strongly dependent on the procedure used for PEC preparation (order of the addition of the PE). 

**Figure 1:**
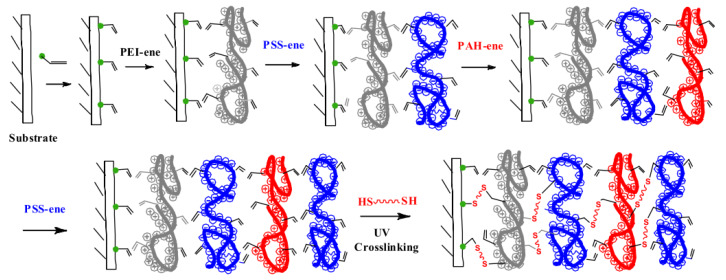
A strategy towards improving stability of polyelectrolyte complex (PEC) films on substrates by cross-linking. Republished with permission of the Royal Society of Chemistry, from [9].

Like all polymers, PEC shows swelling properties when exposed to an appropriate solvent, which affects its permeability and mechanical properties, since the solvent molecules act as plasticizers. As a consequence of uncontrolled swelling, the dissolution of the PEC can occur. Dissolution can be prevented by forming stable three-dimensional structures through the cross-linking of polyelectrolytes (Figure 1), using suitable cross-linkers (e.g., agent containing thiol group) [8,9].

These (cross-linked) materials are called superabsorbent (SAP) polymers or hydrogels because they tend to absorb a solvent rather than to be dissolved in it. The type of bonding in the PEC, among other factors (stoichiometry, polyelectrolytes properties, etc.), mostly depends on the kind of present ionizable functional groups in polyelectrolytes. For example, strong and irreversible electrostatic bonding (e.g., the formation of the ion-pairs), which leads to the high stability of a PEC, can be observed for PECs composed of PEs that contain a quaternary amino, sulphate, or sulphonate group. Strikingly, a significant contribution of non-electrostatic forces to binding energy is observed between the sulphonate group and the primary amino group present in a cationic PE [5]. Additionally, neutral polymers can form PECs with polyelectrolytes due to the formation of hydrogen bonds between them. Finally, two types of bonding (ion-pairing and hydrogen bond) can occur in a PEC containing both copolymer polyelectrolytes and “regular” ones.

Considering the above-mentioned information, it can be deduced that many natural compounds exhibit polyelectrolyte properties. Indeed, many polysaccharides and polypeptides (proteins) are considered polyelectrolytes. As polyelectrolytes, they can form complexes with each other or even with other synthetic polyelectrolytes. Insights into this property have led to a widespread utilization of PEs for the protection (by encapsulation) of biologically active compounds (hormones, peptides, enzymes, and drugs in so called “drug delivery systems”) and their controlled release within a human organism [5,10,11]. Furthermore, the best-known natural polyelectrolytes are nucleic acids.

The property of polyelectrolytes to form various van der Waal’s forces is used for their adsorption to a surface of various nanoparticles (metals or metal oxides, carbon-based nanoparticles, silica, hybrid materials, etc.) or substrates (indium tin oxide (ITO), various carbon materials, metals, Teflon^®^, glass, silica, etc.).

Knowledge of all these PEs properties can be integrated for the production purposes of novel materials, with a possibility to manipulate (via pH and the degree of ionization, ionic strength, temperature, concentration, etc.) their structure, composition, and thickness, which, overall, leads to improvements of their chemical, optical, and electrical properties. The advantages of these materials are used in the development of anticorrosion [12] and antimicrobial coatings [13], fuel cells [14,15], batteries [16], and electrochromic and photovoltaic devices [17]. 

One of the major challenges in the fabrication of sensors is the integration of a sensing element and an electrode substrate (which usually acts as transducer). This integration has to be done in a way so it preserves the properties that are important for the quality of analytical responses. Since many natural polyelectrolytes (of which some are biomolecules), various nanoparticles, luminescent compounds, and inorganic materials can be used as sensing elements, polyelectrolytes fulfil the necessary requirements for the incorporation (immobilization) of various sensing elements. Together with polyelectrolytes’ ability to form thin films on various substrates, it follows that polyelectrolytes are characterized by a huge potential for being used in the design and fabrication of many different types of sensors. A lot of review articles have been focused on the application of polyelectrolytes in chemical and biochemical sensorics. These reviews have mostly covered the role of polyelectrolytes in the development of optical sensing systems [18,19,20,21,22,23]. The main approach in the development of optical sensing systems, based on polyelectrolytes, relies on the utilization of conjugated polymers [24,25] (e.g., conjugated polyelectrolytes). The solubility of these polymers in an aqueous solution is attributed to the presence of charged functional groups. These groups, together with a π-conjugated backbone that exhibits strong absorption or emission of the light, provide platforms for the utilization of these materials in optical sensing systems. The interaction between target molecules and conjugated polyelectrolytes relies on electrostatic interactions, which may have an influence on the optical characteristics of conjugated polymers. Additionally, a great interest in using PEs in the construction of electrochemical sensors has been observed [26,27,28,29,30]. 

In this review, electrochemical sensors that rely on the utilization and role of polyelectrolytes in sensor construction within the last decade is discussed. This includes sensors for various analytes such as metabolites, nucleic acids, gases, humidity, metal ions, and by-products of metabolism. Furthermore, this review covers general approaches to the immobilization of sensing elements and/or transducers (enzymes, nucleic acid, proteins, carbon-based materials, metal or metal oxide nanoparticles (NPs), redox-active metal complexes, bacteria, PEC, etc.), as well as various electrode modification procedures (layer-by layer (LbL), dip-coating, drop-coating, spin-coating, etc.). Many reviews related to electrochemical sensors involving polyelectrolytes, which have mostly focused on certain analytes (DNA) [30], the incorporation of certain sensing elements (enzymes) [29], the use of polyelectrolytes in a certain sensor fabrication method (LbL) [31], in the preparation of the nanocomposite (graphene-polyelectrolytes) for analytical purposes [27], or for the application of carbon nanomaterials in certain sensor architectures [26]; in contrast, this review focuses on the architecture of sensing layer-comprising polyelectrolytes and their role in sensing systems. However, as a keyword, “Nafion^®^” was excluded during database search (Web of Science, ScienceDirect, Scopus, and SciFinder) due to the enormous number of research publications related to sensors that include this polyelectrolyte in their experimental part. “Nafion^®^” has been mostly used as a final layer in sensor architectures due to its characteristic to act as a cation exchanger (e.g., anion excluder) or as a matrix for the entrapment (incorporation/immobilization) of sensing elements (mostly enzymes). 

## 2. Polyelectrolytes in Layer-by-Layer (LbL) Sensor Assembling

### 2.1. Multilayer Film Consisting of Two Components (Layer)

**Scheme 1:**
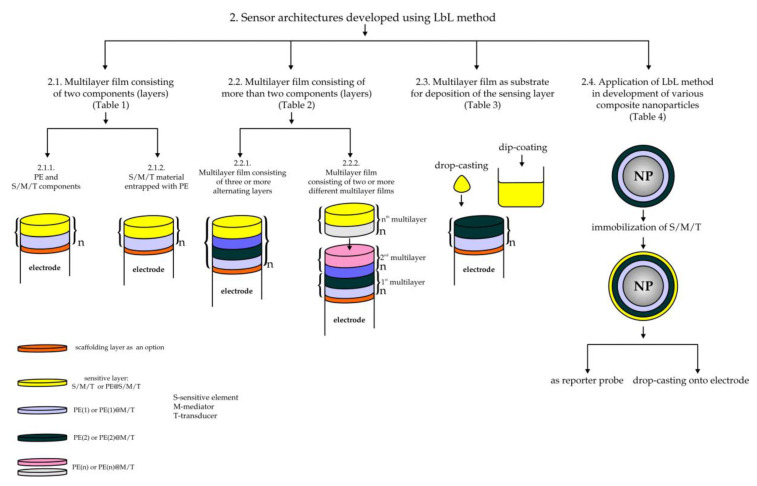
A schematic overview of classification of sensor architectures obtained by using the layer-by-layer (LbL) method featured in articles covered by this review. Hints to the corresponding Tables are also given.

One of the most common methods that utilize polyelectrolytes in fabricating sensors is the LbL method. The ability to manipulate the thickness and composition of a multilayer and the possibility to incorporate, in multilayers, different sensing/mediating/transducing materials with opposite charges to the charge of a polyelectrolyte, makes this method easy, simple, and inexpensive for producing sensing layers. Additionally, a multilayer film can act as an interlayer between an electrode (substrate/transducer) and a sensing layer. In addition, there is a variety of ways to produce ultra-thin structured materials for utilization in electrochemical sensing systems. Additionally, the polyelectrolytes’ feature of being assembled out of different materials (which act as sensing materials, mediators or transducers), with a goal to obtain well-dispersed and homogenous composite materials and the possibility to use such composite materials in LbL assembly, increase in interests in applying this method in sensor fabrication is not surprising. However, the LbL method is characterized by few limitations. These limitations are related to polymer charge density needed for the formation of a multilayer, the disruption of bonds between formed multilayers due to incorporation of various salts and upon change in pH, and, finally, the degradation of the multilayer as a consequence of an inter-polyelectrolyte exchange [5]. In Scheme 1, we propose a principle of the classification of the articles reporting the LbL method in the development of electrochemical sensors.

#### 2.1.1. Sensing Film Consisting of Two Alternate Deposited Layers: PE and a Sensing/Mediating/Transducing Component

The application of the LbL method in various sensor architectures is given in Table 1 (Section 2.1.1). Multilayer films were prepared by the formation of different numbers of alternating layers, and the obtained films were used as sensing parts of a sensor without any further modification. These films were assembled out of two different components that acted as self-containing layers: PE and sensing/mediating/transducing (S/M/T) components. The simplest way to create this multilayer film is to entrap the sensing/mediating/transducing component between (“in sandwich”) two PE layers. Obviously, a sensing/mediating/transducing element should be charged oppositely to the charge of a polyelectrolyte. In addition, several examples of the above-described multilayer films built on solid electrodes that are pre-modified with a scaffolding layer are also given in Table 1.

##### Redox Mediators (Complex Compound) as a Layer

As materials with silsesquioxane structures were used for the electrochemical determination of dopamine, a 3-*n*-propylpyridinium chloride silsesquioxane (SiPy^+^Cl^−^) polyelectrolyte combined with nickel(II) tetrasulphophtalatocyanine (NiTsPc) was used in the formation of a multilayer film for its voltammetric determination. The role of the SiPy^+^ polyelectrolyte is to stabilize NiTsPc because during successive electrochemical measurements, a decrease in current was observed as result of NiTsPc loss [32]. 

A similar procedure was reported for the incorporation of copper tetrasulphonated phtalocyanine dye between poly(ethylenimine) (PEI) layers [33] as a mediator for amperometric detection of mercaptoethanol. In order to facilitate electron transfer toward glassy carbon electrode (GCE) prior to the formation of multilayer film, the authors modified the electrode surface through the drop-casting of the chemically oxidized CNTs. 

The alternating immersion of an ITO electrode into phosphotungstic acid and PEI resulted in the development of an electrochemical sensor for the determination of ascorbic acid and H_2_O_2_ [34]. Entrapping positively charged iron(III)-porphyrin between poly(sodium 4-styrenesulphonate) (PSS) layers for the electrochemical determination of bromate was reported by Lee at al. [35]. Prior to the formation of the multilayer film, a screen-printed carbon electrode was modified by the dispersion of oxidized multi-walled carbon nanotubes (MWCNTs)@Nafion^®^ (note that Nafion^®^ and MWCNTs have the same charge) in order to obtain a negatively charged surface. 

The Dawson type tungstophosphate (POM) was used as a sensing layer for the amperometric determination of nitrite [36]. When the authors deposited poly(allylamine hydrochloride) (PAH) as the first layer in GCE, a low deposition rate of POM was observed. It is important to mention here that the GCE was not chemically or electrochemically treated. To overcome the problem, the authors treated the GCE with aminosilane (ATS) in order to introduce a positive charge to the GCE. In addition, a PSS layer was deposited onto an ATS modified electrode to enhance electron transfer (due to the presence of aromatic rings in PSS) between immobilized POM and the GCE. Then, the LbL method was applied to such a prepared surface via the alternating adsorption of PAH@Trinton-X^®^ and POM. The authors observed that a small amount of surfactant increased the electrocatalytic activity of a film. They attributed such behavior to the presence of aromatic rings in the surfactant, which led to better electron transfer between the immobilized POM and the GCE and/or to an increase in pore size of the film, thus facilitating diffusion (transport) within the film. 

##### Me or MeO NPs as a Layer

The preparation of a composite multilayer film can occur through the alternate deposition of polyelectrolyte matrix and concomitant nanoparticles (NPs). Accordingly, the synthesis of NPs occurs independently of the formation of a composite multilayer film, allowing for the modulation of the synthesis parameters (choice of a reductant and a precursor and their concentration) of the NPs in order to control their shape and size.

An interesting LbL method, involving IrO_x_NPs as a sensing element, for the preparation of a multilayer film was reported by Jović et al. [37]. The authors used the inkjet printing of layers to cast sensing films that were comprised of layers of poly(diallyldimethylammonium chloride) (PDDA) and IrO_x_NPs onto an ITO electrode. Afterwards, the prepared electrode was applied in the potentiometric determination of pH. 

Negatively charged gold nanoparticles (AuNPs) were used as a mediating layer in combination with positively charged polyviologen (PV) [38]. As the first layer was PV, the modification of surface of a gold electrode was needed. To obtain a negatively charged gold electrode, it was necessary to modify it with a molecule that had an atom with high affinity toward the gold (usually sulphur atom) on one side and a functional group that produces an anion upon dissociation on the other side. For this purpose, the authors used sodium 2-mercaptoethanesulphonate. This highly sensing amperometric sensor was used to quantify H_2_O_2_. 

A similar assembly comprised of the polyelectrolyte poly ((2-ethyldimethylammonioethyl methacrylate ethyl sulphate)-co-(1-vinylpyrrolidone) (PQ11) and citrate, which beared a negative charge, stabilized silver nanoparticles (AgNPs) in an ITO electrode. The obtained sensor was applied to the determination of H_2_O_2_ [39].

##### Carbon-Based Nanoparticles as a Layer

Carbon-based materials can be incorporated into a multilayer film as a self-contained layer. In this case, carbon-based nanoparticles should have opposite charges to those of polyelectrolytes. Accordingly, the chemical/electrochemical oxidation/reduction of carbon nanoparticles should be performed before assembly with polyelectrolytes. The main purpose of carbon nanomaterials in this structure is to mediate electrons and/or to increase the charge transfer between a sensing layer and a solid substrate.

For instance, the chemical oxidation of carbon nanotubes [40] or the incorporation of the non-reduced graphene oxide [41] will be performed if a positively charged polyelectrolyte was intended to be used as self-contained layer. In the case when it is graphene oxide, its chemical reduction can be performed within a prepared multi-layered film. Such modified GCEs were used in the voltammetric determination of caffeine and copper(II).

For electrochemical oxidation and the determination of aromatic hydrocarbons, a multilayer film consisting of the conjugated polymer PBCSO_3_^−^ and graphene was cast onto a GCE electrode. The GCE was firstly cast with a layer of electrochemically reduced graphene oxide, probably to improve the basal plane orientation of the multilayer, which should improve the π–π stacking of the aromatic hydrocarbons in the sensing layer [42].

##### DNA as a Layer

A simple LbL approach can be used to obtain sensing multilayer films based on the interaction of nucleic acids with weak cationic polyelectrolytes. In this architecture, besides the primary function, polyelectrolytes have a role as substrates for the immobilization of a nucleic acid probe. 

**Figure 2:**
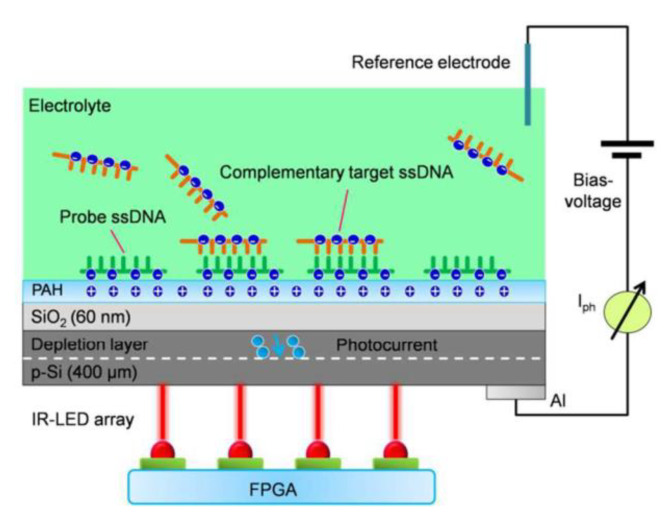
Schematic view of layer structure of a DNA biosensor. Republished with permission of the Royal Society of Chemistry, from [43].

For instance, two similar procedures (Figure 2) were used for the preparation of potentiometric [43] and impedimetric [44] DNA sensors. These multilayer films were cast onto an SiO_2_ substrate (negatively charged at physiological pH) with a DNA probe as the final layer. These sensors were developed as a field-programmable gate array (potentiometric) and field-effect capacitive sensors (impedimetric). 

In addition, the detection of intrinsic molecular charge, induced by the negatively charged phosphate backbone of nucleic acid, was used in the determination of DNA [45]. For this purpose, a multilayer film, consisting of PAH and a DNA probe, was grown onto field-effect chip (SiO_2_). The same procedure was used for the preparation of a capacitive sensor [46]. 

Various multilayer films were prepared by Evtugyn et al. [47] in order to obtain an impedimetric sensor for the detection of DNA damage caused by reactive oxygen species. As platforms for a multilayer film, phenothiazine dyes (methylene blue and methylene green) were applied. 

##### Enzyme as a Layer

As proteins, enzymes (as sensing materials) can also be incorporated as a self-containing layer by the LbL procedure. 

Among several possible LbL modification paths for the modification of a screen-printed carbon electrode (SPCE), one was based on the formation of a sensing multilayer film consisting of PDDA and choline oxidase (ChOx) on an SPCE modified with MnO_2_ (as redox mediator for produced H_2_O_2_) [48]. This sensor was used for the amperometric determination of choline. 

For the amperometric determination of urea in flow mode, a field-effect transistor gate (functionalized with 3-aminopropyl-triethoxysilane (APTES)) was modified with reduced graphene oxide (GO (rGO)). The modified gate was subjected to further modification with negatively charged sodium 1-pyrenesulphonate (SPS). The sensing multilayer film was obtained b they alternate adsorption of PEI and urease [49]. The principle of the operation of this sensor is based on the change in degree of protonation of PEI with increasing pH (as a consequence of an enzymatic reaction). 

A multilayer film, consisiting of sarcosine oxidase and PAH, was used to determine sarcosine [50]. Since the signal of this sensor was based on the generation of photocurrent by cadmium/selenium quantum dots (Cd/SeQDs), the surface of a gold electrode was modified with Cd/SeQDs. For this purpose, the surface was initially cast with 1,4-benzendithiol, after QDs were adsorbed on it. Benzenedithiol has a role of a “glue” (or “bridge”) between a gold surface and Cd/SeQDs via sulphur atoms. In addition, aromatic ring ensures the transfer of electrical signal. After surface modification, the LbL method was used to obtain a sensing multilayer film comprised of sarcosine oxidase. In fact, this sensor was an oxygen sensor, as a decrease of photocurrent (generated at the Cd/SeQDs) is a consequence of the consumption of oxygen in the enzymatic reaction of sarcosine oxidation. 

A similar approach was developed for the construction of an amperometric sensor for the determination of glucose was reported by Tanne et al. [51]. Contrary to the above-presented procedure, the modification of QDs with benzenedithiol was firstly performed, after which the immobilization of such modified QDs at the gold electrode was done. A multilayer film was made from PAH-glucose oxidase. Finally, cross-linking was performed to improve the stability of the multilayer. 

Enzyme-modified field-effect devices, for the determination of penicillin, were also described [52]. The simple architecture of a multilayer consisting of positively charged polyelectrolytes (poly(amidoamine) dendrimer (PAMAM) or PAH) and penicilase was constructed in order to produce an electrochemical impedance spectroscopy (EIS)-based capacitive sensor. Increasing the concentration of penicillin increases the concentration of H^+^ ions (generated during enzymatic reaction), which has an impact on the capacitance–voltage response (the curve is shifted toward more negative voltage). Accordingly, it is necessary to apply voltage from an outsource in order to adjust the working point in the depletion region. 

#### 2.1.2. Sensing/Mediating/Transducing Material Is Entrapped with PE and Applied as a Self-Containing Layer in a Multilayer Film Consisting of Two Alternately Deposited Layers (Repeated Bilayers)

##### Me or MeO NPs–PE as a Layer

As the preparation of stable metal or metal oxide NPs (Me or MeO NPs) solution is a challenge, the preparation methods are mainly based on reduction (mostly chemical) of a metal precursor in the presence of an entrapping (other used terms: encapsulating, dispersing, stabilizing, and assembling) agent that prevents metal particles from aggregating. The most common entrapping agents are PEs, owing to their characteristic of being assembled with charged molecules (precursor ions), resulting in a well dispersed and homogenous mixture. Accordingly, by using PEs in the synthesis of Me or MeO NPs, it is possible to optimize the electrocatalytic properties of the NPs, which is done best through the control of their size and solubility (of metal or metal oxide NPs), as well as their stability (preventing agglomeration). In addition, entrapping Me or MeO NPs can be performed by mixing a solution of PE and the previously synthesized NPs. Furthermore, it is possible to entrap the metal precursor with PE after the preparation of the multilayer film. Finally, the metal precursor in a multilayer film can be a subject of a reduction process. As a result, the formation of MeNPs within the multilayer film occurs.

Of course, the stabilizing (thus dispersion) property of PEs also applies to carbon-based nanomaterials and other sensing/mediating materials. Finally, choosing suitable PEs is also important, since a strong entrapping agent could hamper the access of the species, which is responsible for the electrochemical signal, to the NPs.

Table 1 (Section 2.1.2) presents architectures where a sensing/mediating/transducing material was entrapped with an oppositely charged polyelectrolyte in the first step. The subsequent step is the incorporation of such composite(s), as self-containing layer(s), in a sensing film. 

PtNPs incorporated into the inorganic, positively-charged polymer 3-*n*-propylpyridinium silsesquioxane (PtSiPy^+^) were combined, using the LbL method, with a natural polymer-humic acid to obtain a multilayer film sensitive to 17α-ethynylestradiol [53]. 

The LbL method was used in the incorporation of a metal cation–PE complex into the multilayer film, followed by its chemical reduction [54]. Firstly, a solid substrate was immersed into a (“positive”) suspension of metal cation and chitosan (CS), followed by immersion in a (“negative”) polystyrene sulphonate solution (PSS). The obtained multilayer was exposed to an NaBH_4_ solution in order to reduce present metal cations. The authors used the prepared electrode for the determination of glucose. 

PE-encapsulated AuNPs, prepared by the chemical reduction of the Au(III) with NaBH_4_ in the presence of PEs, were used in assembling multilayer films consisting of AuNPs encapsulated with PAH and AuNPs encapsulated with poly(acrylic acid) (PAA) in order to determine nitrite by cyclic voltammetry (CV) [55]. 

An EIS chip, modified with a multilayer film comprised of PAMAM and CoFe_2_O_4_ encapsulated in PVP, was applied to the impedimetric determination of H_2_O_2_ [56]. In fact, potentiometric analytical response was toward H^+^ ions, produced by the oxidation of H_2_O_2_ at CoFe_2_O_4_ particles 

##### Carbon-Based Nanoparticle–PE as a Layer

**Figure 3:**
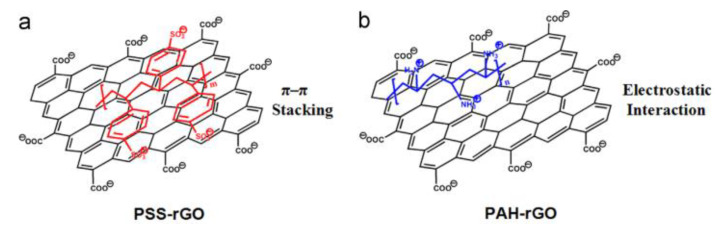
Schematic illustration of two composite materials: (**a**) PSS(−)rGO and (**b**) PAH(−)rGO. Reprinted from [57], with permission from the Elsevier.

Polyelectrolytes–rGO composites and their effective utilization for the fabrication of resistance-based NO_2_ gas sensors were reported by Li et al. [57]. Two PEs (PAH and PSS) were used to modify the rGO after the obtained composites were used in a multilayer film assembly by LbL on a micro-grid array with a pure carbon film (Figure 3). As rGO is difficult to be dispersed in aqueous solutions, the authors dispersed GO in the presence of PEs, followed by its chemical reduction. 

The dispersion of the single-wall carbon nanotubes (SWCNTs) in PDDA for developing new electrochemical transducers, for the determination of a carbaryl pesticide via an enzyme-based layer (acetylcholine esterase-AChE), was described by Firdoz et al. [58]. PDDA was used as a highly positively charged species for the adsorption of the SWCNTs onto a GCE surface and as a layer for the deposition of an enzyme (AChE) using the LbL procedure.

### 2.2. Multilayer Film Consisting of More Than Two Components (Layers)

In order to improve the electroanalytical properties and physical stability of multilayer films, some authors built sensing films consisting of more than two components. The LbL method can be used in the preparation of the final multilayer film comprised of more than two components or comprised of two or more multilayer films. However, it is difficult to divide these architectures according to the respective components, since many of them consist of various materials such as polyelectrolytes, sensing elements (in free form or in a complex with a PE), mediators (redox mediators), and transducers. Here, we classified films according to sensing materials (if it was present in film) incorporated using these two approaches (Table 2).

#### 2.2.1. Multilayer Film Consisting of Three or More Alternately Deposited Layers 

##### Redox Mediators (Complex Compounds) as a Layer 

Three different architected multilayer films were investigated in order to determine dopamine [59]. Two of these were based on a bilayer assembled of PEI and montmorillonite clay or nickel(II) tetrasulphophtalatocyanine (NiTsPc). The third multilayer was constructed by a quadri-layer assembly by the alternate deposition of PEI, MMT, and NiTsPc onto an ITO electrode. A synergistic effect in the quadri-layer of the LbL-prepared film provided an improvement in detection range and a lower limit of detection. 

A multilayer prepared by the alternate deposition of rGO, PAH, and Prussian blue onto a gold electrode anchored with PEI was reported by Pajor-Świerzy et al. [60]. The multilayer film was formed by the LbL method and applied to the detection of H_2_O_2_.

Conductive polymers were usually utilized in this sensor architecture. The electroactive multilayer film of polyelectrolytes and Prussian blue nanoparticles was applied to H_2_O_2_ sensing [61]. To obtain a positive charge, a layer of PEI was firstly placed onto a gold electrode surface, after which the multilayer film was grown by Prussian blue nanoparticles and poly(3,4-ethylenedioxythiophene) (PEDOT):PSS in its conducting state. PEDOT:PSS was applied within a potential region where the Prussian blue exhibited good electrocatalytic activity. Accordingly, a synergistic effect was observed. PSS had a role as the anionic polyelectrolyte dopant.

A similar approach was reported by Pajor-Świerzy et al. [62]. A gold electrode was covered with layers of PEI, Prussian blue conductive polymer (poly(pyrrole) or poly(aniline)), and PAH. Such a designed electrode was used for the amperometric determination of H_2_O_2_. 

In addition, a multilayer film deposited by alternate immersion in positively charged PDDA, a negatively charged solution of a PAA–MoS_2_ composite, then a positively charged solution of a PAM–MoS_2_ composite, and finally a negatively charged solution of PEDOT:PSS, onto various substrates, was reported [63]. These sensors were applied in the amperometric determination of H_2_O_2_.

##### DNA as a Layer

As mentioned before [47], in order to obtain an impedimetric DNA sensor, authors have used a variety of LbL architectures. Three-layered films were prepared so that they included DNA. The films consisted of PSS, PAH, and DNA layers deposited onto a GCE (modified with methylene blue or green) in a different order.

##### Enzyme as a Layer

Lactate oxidase (LOx) was embedded in multilayer polyelectrolyte film consisting of PDDA, AuNPs, and LOx onto a thiolated (with a negatively charged-3-mercapto-1-propane sulphonic acid sodium salt (Na-MPS)) polycrystalline Au electrode [64]. This simple construction was used for the amperometric determination of lactate.

#### 2.2.2. Multilayer Film Consisting of Two or More Multilayer Films

##### Me and MeO NPs as a Layer 

The utilization of the LbL method in flow mode for the preparation of microelectrodes for application in microfluidic devices was reported by Kumlangdudsana et al. [65]. The first multilayer film (obtained by LbL) of the poly(diallyldimethylammonium chloride) (PDADMAC)-PSS was deposited onto a channel in order to enhance adhesion between gold nanoparticles and a substrate. The second film was deposited onto the first film and included PDADMAC and AuNPs. All these layers were prepared in a flow mode. To demonstrate the usefulness of the prepared conductivity sensor, the authors tested the microelectrode for the detection of KCl in a solution. However, the authors revealed the potential utilization of these electrodes in microfluidic devices for the determination of various species based on the change in conductivity. 

The fabrication of LbL films based on the functionalized rGO (graphene) nanopellets and AuNPs for the detection of pesticide-methyl parathion was reported by Rodrigues et al. [66]. The used rGO (graphene) nanoplatelets were stabilized in PDDA or PSS to obtain a positive or a negative charge of the rGO, respectively. Two multilayer films were produced by the LbL procedure: The first one was produced by the alternate deposition of rGO stabilized with PDDA and rGO stabilized with PSS, and the second one was prepared by the formation of a multilayer film comprising AuNPs and rGO stabilized with PSS. 

As the SiO_2_NP layer on top of the semiconducting layer plays a role of a charge collector, it can have effect on the conductance of the semiconducting layer deposited at the gate of ISFET (ion-selective field-effect transistor). Together with the tunable electrochemical properties of a nanomaterial thin-film, this architecture could be used to develop a variety of electrochemical (bio)sensors [67]. The authors used three films prepared by the LbL procedure. A PDDA-PSS film was produced as the first film and had a role to enhance the surface charge for functional nanomaterial thin-film. The second film was produced from PDDA and SWCNTs or In_2_O_3_, while the third film comprised PDDA and SiO_2_NPs. This sensor was applied to the determination of pH by measuring changes in chemoresistance.

##### Carbon-Based Nanomaterials as a Layer 

**Figure 4:**
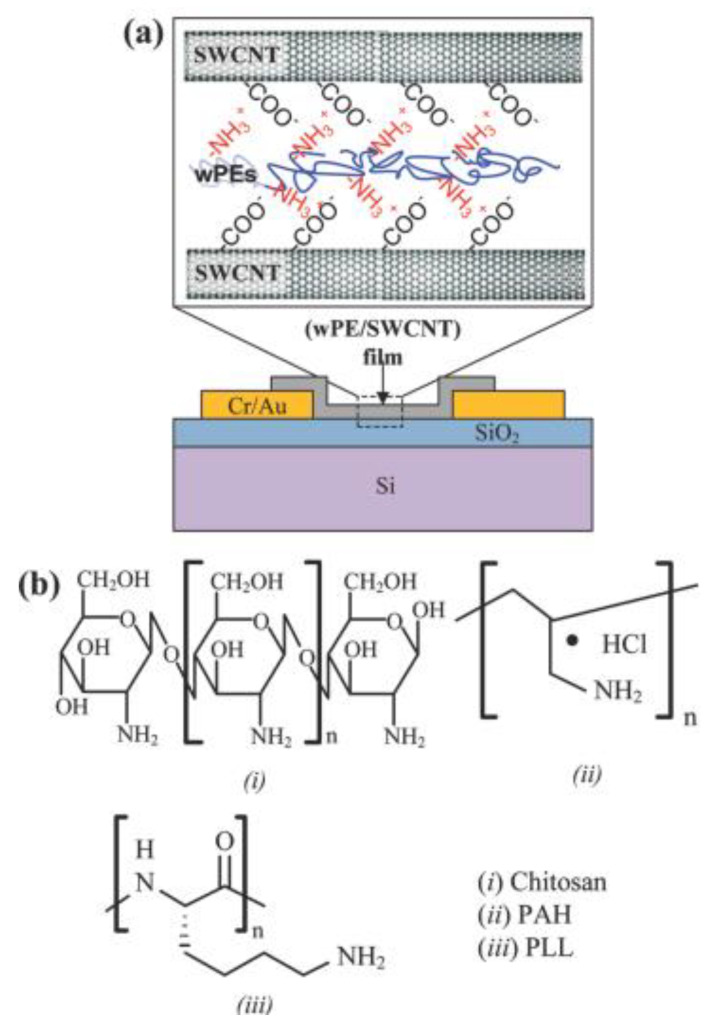
Layer-by-layer-assembled carbon nanotube thin-film device. (**a**) schematic of device and (**b**) molecular structure of the polyelectrolytes. Reprinted with permission from [68]. Copyright (2011) American Chemical Society.

Carboxylated SWCNTs were assembled via the LbL procedure with a weak polyelectrolyte possessing amine-functionalized groups with different dissociation constants that controlled the number of proximal H^+^ and OH^−^ (PAH, PDDA, and chitosan). The concentration of these ions influences the conductivity of SWCNTs films, so they can be used to measure pH [68]. The authors used an assembly of the two films that consisted of PDDA-PSS and chitosan-SWCNTs (Figure 4). 

##### Enzyme as a Layer 

**Figure 5:**
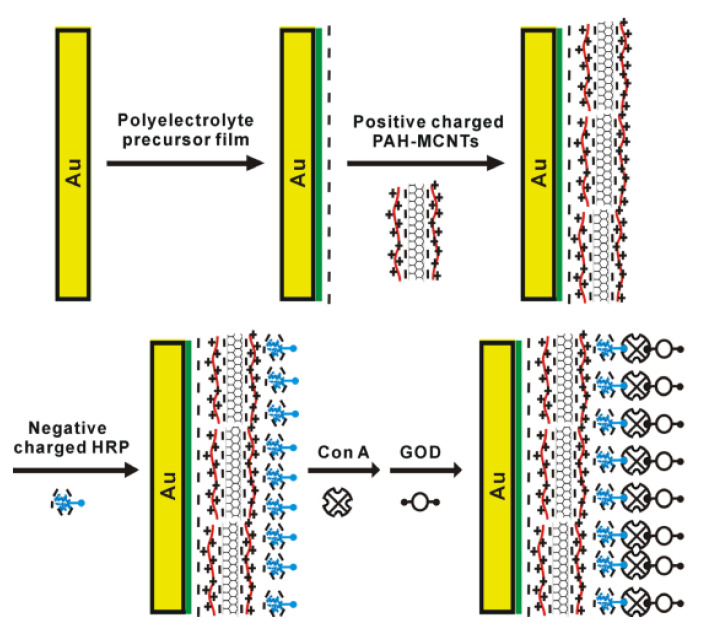
Schematic illustration of construction of a bi-enzyme bio-nano-multilayer electrode based on functionalized CNTs and sugar–lectin bio-specific interactions. Reprinted from [69] with permission from the Elsevier.

Three multilayer films cast using the LbL procedure onto a gold electrode modified with mercapto sulphonic acid in order to obtain a negatively charged surface were used for the determination of glucose [69]. A precursor bilayer film (PAH-PSS) was cast on a modified gold electrode (by MPS) in order to enhance the surface charge of the functional nanomaterial film. On the precursor film, the LbL method was applied in order to obtain a film consisting of a composite (PAH with MWCNTs) and a negatively charged horse radish peroxidase (HRP) (Figure 5). Secondly, the LbL method was used for the preparation of a film consisting of Concanavalin A (ConA) and GOx. ConA has a bridging role between two enzymes due to its bio-specific binding properties to HRP and GOx (via a lectin–sugar interaction). 

For the determination of pH and acetylcholine, a thin-film solid-state electrode based on AChE and oxidized SWCNTs, was reported [70]. Through the hydrolysis of acetylcholine (to carboxylic acid and choline), local pH change and the collection of generated protons in the vicinity of the SWCNT film were observed. This induces a change in the electric potential of the working electrode. This platform can be used as a pH electrode and used for further modification for obtaining various (bio)sensors. A thin-film pH solid-state electrode was assembled out of three different multilayer films comprising PDDA-PSS, PDDA-carboxylated SWCNTs, one layer of PDDA (to obtain a positively charged layer), and PSS-AChE.

### 2.3. Multilayer Film as a Substrate (“Precursor” Film) for Deposition of a Sensing Layer 

A polyelectrolyte multilayer film can be deposited as a “precursor” film onto a substrate, after which various immobilization methods of a sensing element can be applied. The role of the polyelectrolyte multilayer is usually to minimize interference of the substrate and to provide a more uniformly charged surface. Additionally, it is believed that this precursor multilayer film can facilitate nanoparticle deposition. In addition, this film can be prepared in such a fashion that it contains various mediating species (e.g., redox mediating compounds, metal or metal oxide nanoparticles, and carbon-based nanomaterials) that can facilitate electron transfer toward an electrode and can be used for tuning the electrode working potential. These architectures are presented in Table 3.

#### 2.3.1. Redox Mediators (Complex Compounds) in a “Precursor” Multilayer Film

**Figure 6:**
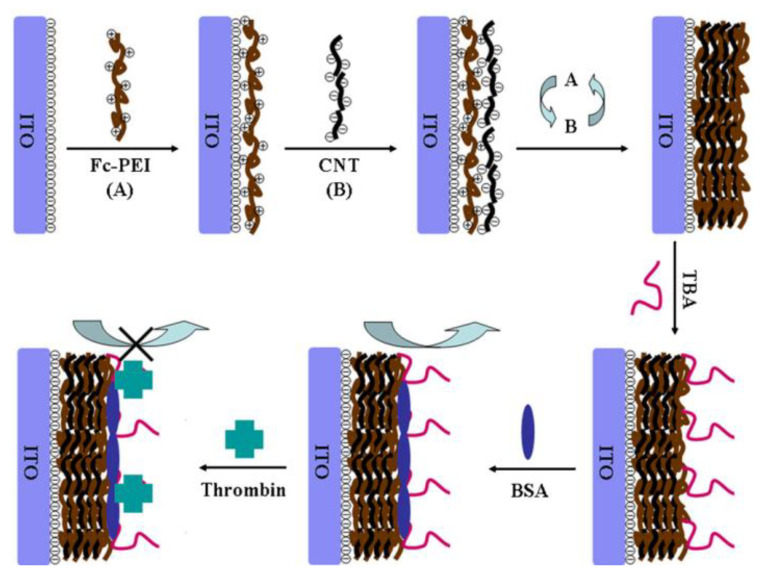
Schematic diagram of electrochemical aptasensing strategy based on the LbL method in the detection of thrombin. (**A**) immobilization of the ferrocene entrapped in PEI and (**B**) immobilization of carbon nanotubes. Reprinted from [71], with permission from the Elsevier.

An LbL polyelectrolyte multilayer can be used as a substrate to embed a nucleic acid or aptamer whilst maintaining their affinity and specificity for the cognate target (Figure 6). Aptamers for the detection of thrombin and lysosomes were deposited onto an ITO electrode that was modified with ferrocene (Fc). Ferrocene was entrapped in PEI and used with the LbL method to prepare a multilayer film with CNTs, after which the drop-casting of the appropriate aptamer was performed. Finally, to reduce non-specific bonding, a bovine serum albumin (BSA) solution was dropped onto the sensing layer. The electrochemical signal of Fc (obtained by differential pulse voltammetry (DPV)) was decreased in the presence of a target analyte [71]. 

A similar procedure was used to develop a sensor for chiral peptides [72]. Negatively charged ITO was covered with a film, using the LbL procedure, consisting of a redox mediator methylene blue (MB) entrapped together with PDDA and an rGO (graphene)–PSS composite. The graphene multilayer plays significant role in this sensing architecture: it allows for the accumulation of MB, facilitates electron transfer, and provides an adsorption site for the aptamer. Aptamer was incorporated by simple drop-casting procedure onto a precursor multilayer film. To prevent non-specific adsorption, the surface of the sensor was treated with BSA.

The LbL method was applied to the incorporation of a redox mediator (NiTsPc) onto fluorine doped tin oxide slides (FTO) by alternate deposition with SiPy [73]. After the formation of the mediating multilayer film, additional SiPy was deposited onto the film in order to obtain a positively charged surface in order to deposit enzyme (GOx) by drop-casting. In order to improve the stability and selectivity of the prepared sensor, a Nafion^®^ solution was applied to the enzyme layer. This sensor was used for the amperometric determination of glucose.

#### 2.3.2. Me or MeO NPs in a “Precursor” Multilayer Film

An ITO electrode, modified with negatively charged PSS, was used as a substrate for building a multilayer thin film consisting of a positively charged MWCNTs–PDADMAC composite and AuNPs. This film was used as a precursor film for the drop-casting of glutathione (GTH). GTH was used to improve electrocatalytic activity toward dopamine [74]. 

**Figure 7:**
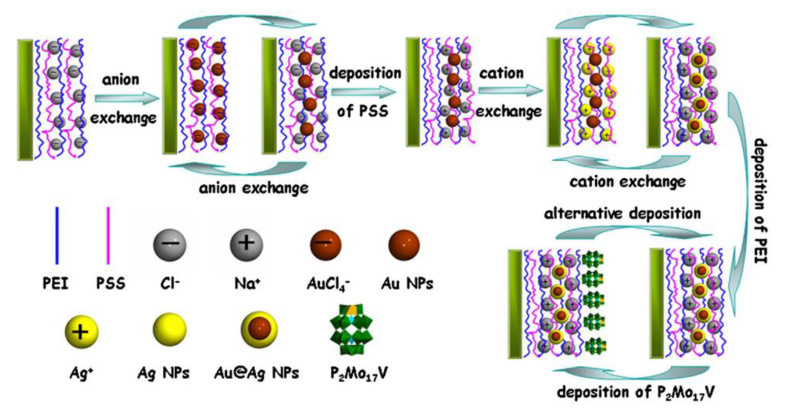
The schematic illustration of a composite film containing Au@2Ag core-shell nanoparticles. Reprinted from [75] with permission from the Elsevier.

A PSS-PEI precursor film, with an additional final PEI film, was deposited onto an ITO electrode, after which the electrode was exposed to AuCl_4_^−^. The reduction of the adsorbed Au (III) species was performed with NaBH_4_. A prepared layer of AuNPs was exposed to a solution containing Ag^+^, followed by Ag^+^ reduction using ascorbic acid. This procedure resulted in the formation of Au@2Ag core-shell nanoparticles at the formed polyelectrolyte multilayer (Figure 7). This structure was immersed in a P_2_Mo_17_V solution containing PEI. The role of the Au@2Ag core-shell nanoparticles was to enhance the activity, selectivity, and stability of a sensor through the synergistic effect of bi-metallic electrocatalysts. The prepared sensor was successfully used in the amperometric detection of cysteine [75].

An amperometric immunosensor for the determination of the apolipoprotein APOE-4 was developed using the immobilization of anti-APOE-4 onto fractal gold (FracAu) [76]. For producing FracAu, an ITO electrode was modified using the LbL method with PDDA and PSS. This precursor layer was exploited for the potentiostatic electrodeposition of FracAu in a solution containing Au(III). For a highly sensing sensor, the authors used the sandwich approach with horse radish peroxidase-labelled anti-APOE-4. Additionally, hydroquinone (added to solution as a redox mediator) was used in order to monitor the reductive current. The authors concluded that combination of a gold fractal nanostructure and enzyme-catalyzed signal amplification enabled the sensor to obtain better sensitivity with lower detection limits. 

Differential pulse voltammetry was used for the detection of targeted DNA [77]. Selectivity was obtained through the immobilization of a DNA probe onto AuNPs. However, AuNPs were deposited onto a PE multilayer film of PAH-PSS, which was grown at the Au electrode surface modified with mercaptopropionic acid (MPA). 

#### 2.3.3. DNA in a “Precursor” Multilayer Film

The immobilization of the AChE enzyme through the non-covalent immobilization (by drop-casting) of the enzyme solution onto a previously modified GCE for the determination of reversible AChE inhibitors was reported by Davletshina et al. [78]. This immobilization was based on weak electrostatic inter-molecular interaction between a PE multilayer film prepared using the LbL method and the enzyme. Furthermore, donor–acceptor interactions and hydrogen bonds contributed to the stability of the enzyme layer. These physical interactions had a small influence on the steric structure of the enzyme. This influence is important when it is necessary to detect reversible inhibitors, as the interaction between inhibitors and enzymes takes place outside of the enzyme’s active site. The authors deposited PE film using the LbL method (PAA-PSS or PAA-DNA) onto a GCE modified with carbon black and cobalt phtalocyanine (redox mediator). DNA was used in the polyelectrolyte film as a strong polyanion with the ability to consolidate complex reactants. 

#### 2.3.4. Unmodified PEs as a “Precursor” Multilayer Film

The same group of authors used a similar approach for the detection of AChE inhibitors, donepezil, and berberine [79]. The sensor was based on hampering the hydrolysis of thiocholine by inhibiting AChE. For the determination of thiocholine, cobalt phthalocyanine was applied in combination with carbon black as the first layer onto a GCE; afterwards, the LbL method was applied to produce a precursor multilayer PAA-PSs. AChE was deposited on such a prepared layer by drop-casting. In some cases, an additional polyelectrolyte layer was deposited on the enzyme layer for its protection. As an analytical signal, the decrease in anodic current was monitored. 

Among several different LbL modification paths used for the modification of a screen-printed carbon electrode (SPCE) in the work of Dontsova et al. [48], one was based on the formation of a precursor multilayer film consisting of PDDA and PAS. MnO_2_ was applied as a redox mediator onto the surface of the SPCE in order to oxidize H_2_O_2_ produced by choline oxidase. ChOx was deposited on the PE multilayer film by drop-casting. As mentioned before, the authors determined choline with amperometry. 

The polyelectrolyte film used in the work of Lakard et al. [80] was composed of two biopolymers: a negatively charged carboxymethyl pullulan and a positively charged chitosan. The urease was covalently immobilized onto a layer of the multilayer film (the final layer was carboxymethyl pullulan) through a carbodiimide coupling reaction. Simultaneously, a polysaccharide multilayer film was crosslinked through a reaction between amine groups of chitosan and the carboxylic acid moieties of carboxymethyl pullulan. Consequently, a highly stabilized multilayer film was obtained. Together with the architecture based on the physical adsorption of the enzyme onto the polyelectrolyte multilayer film, the obtained sensors were tested for the potentiometric determination of urea. 

**Figure 8:**
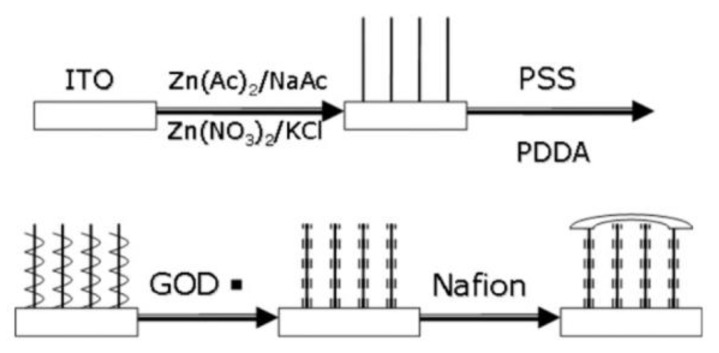
Schematic diagram of self-assembled GOx onto ZnO nanorod arrays synthesized by electrodeposition on ITO. Reprinted from [82] with permission from the Elsevier.

The same architecture was used for the potentiometric determination of H^+^ ions and urea, though on a different substrate [81]. In the above-mentioned work, the substrate was polyaniline (PANI)-modified carbon, while in the latter, it was PANI-modified SPCE. An interesting role of PEs in sensor architectures was reported by Zhai et al. [82]. The authors electrodeposited ZnO nanorods (ZnONRs) or nanoparticles (ZnONPs); afterwards, they used a multilayer film consisting of PSS and PDDA as a support to highly vertically oriented ZnONRs (Figure 8). On such surrounded ZnONRs, GOx was immobilized on the outer PEs layer through physical adsorption. Finally, Nafion^®^ was applied on the prepared sensing layer. This sensor was used for the amperometric determination of glucose.

### 2.4. Application of the LbL Method in Development of Various Composite Nanoparticles (Nanospheres, Hollow-Shell Particles, Reporter Probes. etc.) and Other Sensor Architectures

#### 2.4.1. Composite Nanoparticles (Nanospheres, Hollow-Shell Particles, Reported Probes)

As presented above, gold and silver nanoparticle layers, decorated with nucleic acids or proteins, were used in various electrochemical detection systems, as sensing and transducing layers. These materials display unique or improved electroanalytical properties due to the synergistic activity of both components. Furthermore, the functional integration of nucleic acids or proteins and other nanoparticulate materials can be achieved using a polyelectrolyte multilayer film (or a modified version of a film with metal or metal oxide nanoparticles) deposited on the surface of the nanoparticulate material. These materials are characterized by predefined and biocompatible cargo-carrying and targeting capabilities, which means they can be used in the preparation of sensing layers at the solid-state electrode surface or in the preparation of competitive particles or labelled particles for the competitive or “sandwich” method, respectively. A summary of these applications is shown in Table 4.

A thiolated DNA probe captured on a gold electrode was used for the determination of targeted DNA by a competitive method [83]. Competitive particles were prepared by the immobilization of biotin onto the complementary DNA. After hybridization occurred, streptavidin-functionalized AgNP-tagged carbon nanosphere (CNS) particles were introduced in the system and a bond, by means of an interaction between captured biotin and streptavidin, occurred. Accordingly, if the targeted DNA in sample was present, a decrease in the linear sweep voltammetry (LSV) signal occurred. Streptavidin-functionalized AgNP-tagged CNS was prepared using the LbL method by covering the CNS with a multilayer film consisting of PAH and PSS. Such modified CNS was introduced in solution of Ag^+^ ions and citrate, respectively, which resulted in covering of CNS with AgNPs. At the surface of the AgNPs, streptavidin was immobilized by mixing solution of streptavidin and AgNP-tagged CNS. 

Additionally, polyelectrolytes can be used in the preparation of hollow-shall capsules, using polystyrene-co-acrylic acid (PSA) particles as templates [84]. The deposition of four PAA-PSS bilayers onto PSA spheres was performed using the LbL method, after which tetrahydrofuran (THF) was used to remove PSA. The hollow-shell PAA-PSS capsules were loaded with Au(III) (in the presence of detergent—cetyl trimethyl ammonium bromide (CTAB)), as it was expected that negatively charged AuCl_4_^−^ ions interact with the positively charged PAA. After loading, reduction with ascorbic acid was performed to obtain AuNPs. The role of the hollow-shall PAA-PSS loaded with AuNPs capsules was for them to serve as electrochemical labels attached (via Au-S bond) to modified magnetic Fe_2_O_3_ particles. Fe_2_O_3_ particles were modified with a DNA probe. After modified AuNPs and modified Fe_2_O_3_ particles were bonded, magnetic collection and separation could be performed. Finally, quantification was performed by differential pulse anodic stripping voltammetry (DPASV) after the dissolution of the capsules in acid. The high metal content of labels, together with magnetic collection, provided the low limit of detection. 

The same principle of the immobilization of enzymes (AChE or HRP) was used in order to determine pesticides (inhibitory mechanism) or H_2_O_2_ [85]. Polystyrene sulphonate (PSS) was used as a template for the preparation of PAH-PAA or PEI-PAA polyelectrolyte microspheres. The enzyme was immobilized onto the surface of microspheres. The enzyme-modified microspheres were cast onto the activated surface of SPCE. PAA-terminal microspheres were used for the immobilization of AChE (total charge of the microsphere is negative owing to the charge of PSS), while PEI-PAA was favored for the immobilization of HRP, probably due to enhanced hydrogen bonding and a favorable enzyme conformation. The authors concluded that the immobilization of AChE on a negatively charged multilayer film results in an increased sensitivity due to an optimal enzyme conformation, as well as in a decreased stability due to electrostatic repulsion. In the case of HRP (positively charged), immobilization on a negative multilayer film enhances stability though electrostatic attraction, while, at the same time, providing favorable enzyme conformation. 

The electrochemical detection of DNA hybridization was investigated using PSA poly-beads, modified with the negatively charged mercaptoacetic acid-capped CdTeQDs [86]. Firstly, PSA polybeads were entrapped together with a multilayer of PAH and PSS. The outer PAH layer ensured that poly-beads had a smooth, uniform and positively charged surface, which facilitated the deposition of the negatively charged mercaptoacetic acid-capped CdTeQDs. These CdTe-tagged poly-beads possessed a good biocompatibility, good solubility, and good dispersion properties, as well as size-tailored properties. In addition, the authors modified QDs with streptavidin to ensure coupling with a biotin labelled DNA(2) probe. This DNA probe was supposed to be located at the surface of the electrode, owing to hybridization with a previously hybridized DNA target. This DNA target was hybridized with an immobilized DNA(1) probe onto a glass substrate modified with glycidoxypropyltrimethoxysilane (GPTMS) via the NH_2_ group of the DNA(1). By using these polybeads as a label in the sandwich procedure, the electrochemical detection of DNA hybridization, through the coupling of highly sensing stripping voltammetry measurements, was developed. 

The production of a reporter DNA probe on PSA particles via the conjugation of the DNA probe onto the previously adsorbed AuNPs, was reported by Kuan et al. [87]. The AuNPs were adsorbed onto PSA modified with PAA and PSS using the LbL method. The DNA probe was immobilized onto SPCE by electrostatic adsorption. After hybridization with the targeted DNA, a reporter labelled-DNA probe was added to the solution; afterwards, the second hybridization took place on the non-hybridized part of the targeted DNA (sandwich method). As an electroanalytical method, DPASV was chosen.

#### 2.4.2. LbL Procedure in Other Sensor Architectures

**Figure 9:**
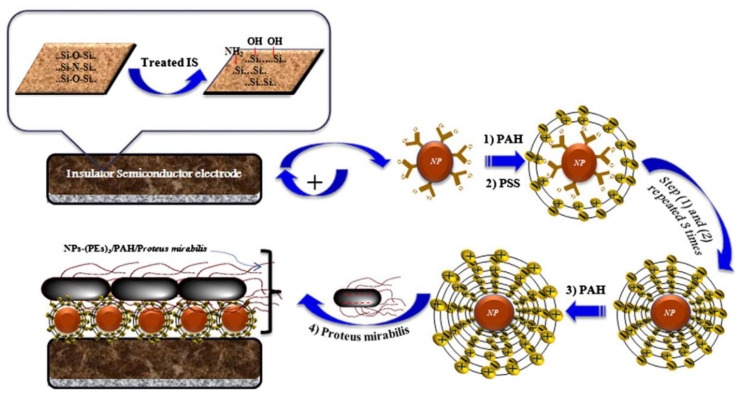
Schematic view of a capacitive urea biosensor based on Fe_3_O_4_ and polyelectrolytes. Reprinted from [88] with permission from the Elsevier.

AuNPs were alternately coated with PEs (PAH and PSS), with the last layer being PAH (positive charge). Such modified AuNPs were deposited onto a Si/SiO_2_ substrate by drop-casting, followed by the drop-casting of bacteria (proteus mirabilis). Finally, glutaraldehyde vapor was applied over the sensor in order to allow for the reticulation of bacteria (e.g., cross-linking of bacteria) This sensor (Figure 9) was used in the potentiometric determination of urea [88]. 

**Figure 10:**
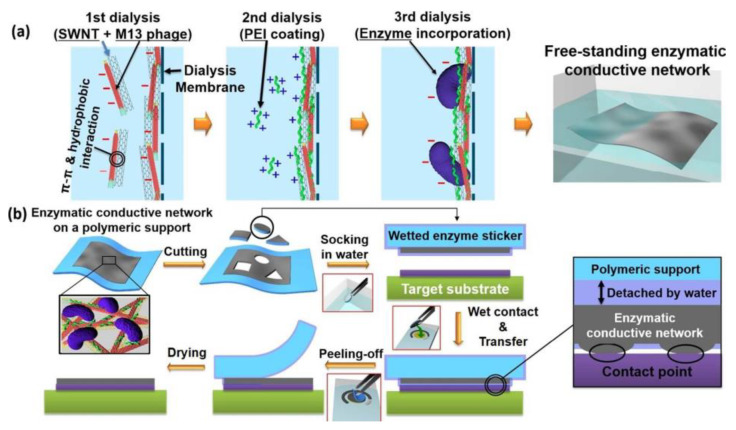
(**a**) Illustration of hydrodynamic layer-by-layer assembly of enzymatic conductive nano-networks. (**b**) Structure of an enzyme sticker and principles exploited for the contact-printing of an enzymatic network film. Reprinted with permission from [89]. Copyright (2018) American Chemical Society.

Lee at al. [89] used a negatively charged M13 phage (anionic polyelectrolyte) as a biological glue to entrap SWCNTs via π–π stacking. They created a multilayer on the cellulose acetate polymeric membrane using sequential dialysis in solutions that contained M13–SWCNTs composites, a PEI solution, and a solution of GOx. This was named an enzyme sticker (Figure 10). The sticker can be transferred to electrode systems (screen printed Au or sputtered Pt electrode), via the “wet” contact. This can be done due to the interaction between the sticker and cellulose membrane being weaker than the one between the sticker and working electrodes. This system was used for the amperometric determination of glucose.

A polyelectrolyte multilayer film can be used as a protective layer for an enzyme, as reported by Buron et al. [90]. The authors used SPCE modified with electropolymerized PANI for the immobilization of urease by casting an aqueous suspension of the enzyme onto the surface of SPCE. This structure was fortified with a polyelectrolyte multilayer film prepared by the alternate immersion of the electrode into chitosan and carboxymethylpullulan (CMP) solutions.

A film consisting of electrodeposited PDDA and negatively charged CNCC was produced using the LbL method in order to form a stable scaffolding multilayer for the electropolymerization of EDOT [91]. The prepared electrode was applied to the amperometric determination of nitrite.

## 3. Polyelectrolytes in Casting/Coating Methods

**Scheme 2:**
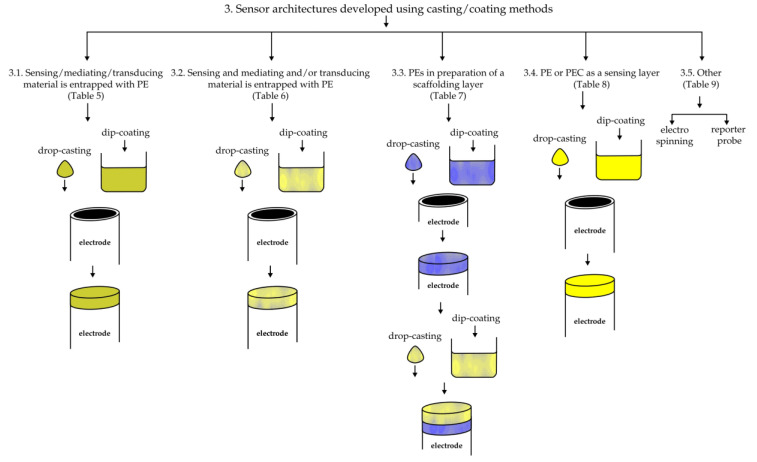
Classification of sensor architectures based on casting/coating methods in articles discussed in this review, together with hint to the respective tables.

Among a number of techniques used for the deposition of thin film layers onto a solid substrate, drop-casting has attracted a lot of attention due to its simplicity, little material waste, and fast preparation. However, some drawbacks, such as poor uniformity, a low control of the layer thickness, and limitations in coverage area, have to be taken into consideration; since this correlates with the electrochemical behavior of the prepared layer. To overcome some of these drawbacks, drop-casting, in synergy with a spin of the substrate—so called spin-coating—provides a more uniform film thickness compared to drop-coating. Dip-coating can provide a very uniform and close-packed film, but many variables should be optimized for the preparation of a satisfying film. In Scheme 2, the principle of the classification of articles, including casting/coating methods in the development of electrochemical sensors, is presented.

### 3.1. Sensing/Mediating/Transducing Component Is Entrapped with PE 

As polyelectrolytes can transfer their surface charge to neutral or oppositely charged electroactive materials, as well as to sensing materials to enable them to be dispersed, it is not surprising that many authors have used the drop-casting or dip-coating of these mixtures for the modification of electrode surfaces. In Table 5, a sensing layer obtained by the drop-casting or dip-coating methods in a simple architecture—entrapping a sensing material (or redox mediator) in a polyelectrolyte matrix—is present.

#### 3.1.1. Redox Mediators (Complex Compounds)–PE Composite

A water solution of Prussian blue, stabilized with cationic poly(dimethydiallylammonium chloride (PDMDAAC), was used in the dip-coating modification methods of an ITO electrode [92]. The obtained micro-gap sensor was used for the impedimetric determination of water in organic solvents. 

**Figure 11:**
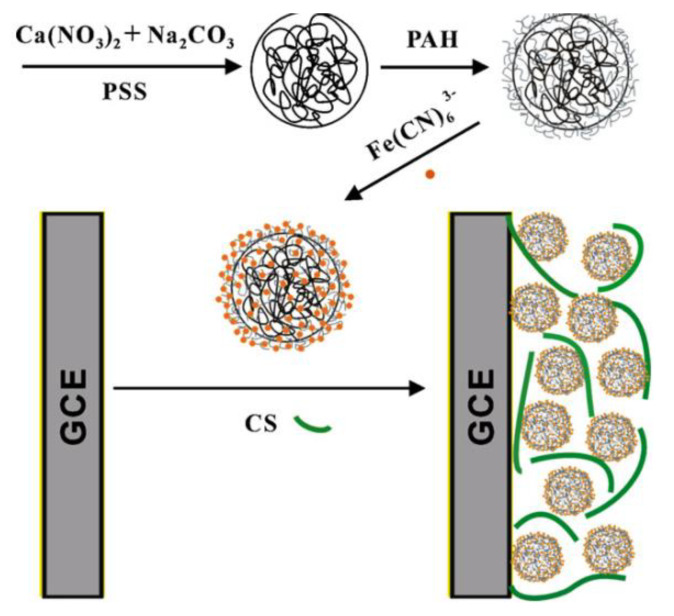
Schematic presentation of the fabrication of ascorbic acid sensor based on porous PE^−^CaCO_3_ microspheres containing an Fe(CN)_6_^3−^ ions modified GCE. Reprinted from [93] with permission from the Elsevier.

To increase the density and distribution of the Prussian blue, Li et al. [93] deposited it onto a CaCO_3_ microsphere modified with PSS and PAH. Firstly, microspheres were prepared in the presence of PSS (as a dispersing agent and the first layer), after which such a prepared material was covered with PAH. To obtain a multifunctional material, the authors dispersed PE-modified microspheres in a solution containing Fe(CN)_6_^3−^ (Figure 11). This multifunctional material was additionally dispersed in a solution of chitosan to obtain the final layer. The above-mentioned suspension was placed on the surface of a GCE, and the prepared electrode was used for the amperometric determination of ascorbic acid.

The stabilization of ferrocenium ions in various PE matrixes by ionic interaction and its application in NO detection was reported by Mathi et al. [94]. The ferrocenium ions were prepared by the oxidation of ferrocene in diethyl ether, followed by extraction with a polystyrene sulphonic acid (PSS) or a Nafion^®^ solution. Prepared matrices were used in the modification of a GCE. 

#### 3.1.2. Me or MeO NPs–PE Composite

Prepared AgNPs, in the presence of N1-(3-trimethoxysilylpropyl)diethylenetriamine (TPDT) (as a reducing agent) and PADA, were found to be catalytically active in the electrochemical reduction of H_2_O_2_ and the catalytic conversion of 4-nitroaniline [95]. The reduction of Ag^+^ ions was performed in the presence of PADA as a capping agent. Moreover, in addition to its role of a reducing agent, TPDT has a role as a capping agent. The prepared composite was used for the modification of a GCE by drop-casting. The authors found out that PADA improved the catalytical behavior of AgNPs. 

Wrapping the stabilized AgNPs with sodium dodecyl sulphate (SDS) (as dispersant) by PAA and application of the prepared suspension for the modification of a screen-printed graphite electrode in order to obtain a sensor for the voltammetric determination of thiourea was reported by Pedre et al. [96]. 

The preparation of bi-metallic Au/Ag nanoclusters (NCs) embedded into the PADA matrix and the modification of a GCE by drop-casting in order to obtain an NO selective electrode, as reported by Viswanathan et al. [97].

#### 3.1.3. Carbon-Based NPs–PE Composite

The dispersion and incorporation of carbon-based nanomaterials in a PE(s) matrix and the application of obtained suspension, dispersion, or solution in the modification of an electrode surface became an easy, inexpensive, and versatile method for the production of sensing layers. However, some inherent problems regarding the stacking of a substrate and a modified layer can reduce the conductivity and lead to interference from the conductive substrate.

A nanohybrid consisting of MWCNTs and chitosan was immobilized onto the surface of a gold electrode by drop-casting [98]. This nanohybrid had a role of a “conductive bridge” between two separated gold surfaces, printed on an alumina substrate. This resistance-based sensor exhibited excellent an analytical performance in humidity sensing. 

The suspension of an MWCNT composite reinforced with sulphonated poly(3,4-ethylenedioxytiophene) (S-PEDOT) was utilized in the voltammetric determination of nitrobenzene [99]. The authors functionalized a conducting polymer (PEDOT) with a sulphonate group to obtain a conjugated polyelectrolyte, with a goal of achieving good conductance and polyelectrolyte properties. 

A simple procedure of mixing S-PEDOT and MWCNTs to obtain a stable suspension that was drop-cast onto a GCE was reported [100]. A simple method, based on entrapping MWCNTs into polyelectrolyte copolymer poly(2-hydroxyethyl methacrylate)-b-poly (2-(dimethylamino) ethyl methacrylate (PHD) (positively charged due to presence of protonated dimethylamino group), followed by the drop-casting of suspension onto the GCE was applied. The nanocomposite films that combined the electrocatalytic properties of MWCNTs and the electrostatic attraction of PHD was used as a sensing layer for the amperometric determination of bisphenol-A. 

The investigation of small carbon nano-onions, in conjugation with PDDA, revealed that this composite could be used for the electrochemical determination of dopamine in the presence of ascorbic and uric acid [101]. As the most commonly used polyelectrolyte, Nafion^®^ was applied as a matrix to entrap carbon-based materials. 

Sensing the voltammetric detection of trace elements (Pb and Cd) in real (water) samples, using a surface-modified GCE with oxidized MWCNTs entrapped in Nafion^®^, was reported by He et al. [102]. Intriguingly, the authors did not explain nature of the conjunction between the negatively charged Nafion^®^ and the oxidized (negatively charged) MWCNTs. 

A novel and simple electrochemical sensor for the determination of 4-nitrophenol, based on a PDDA-rGO (in article termed as graphene) film placed onto a GCE, was reported by Peng et al. [103]. PDDA-rGO represented a reduced composite made in a chemical process (including hydrothermal process for the reduction of GO). 

A homogenous mixture of GO and positively charged PDDA or negatively charged PSS was treated with hydrazine in order to obtain a composite of PE and rGO. A gold electrode was dip-coated in the dispersion of rGO within the solution of PE to obtain a humidity-sensing layer [104]. 

#### 3.1.4. Other–PE Composite

Finally, a whole biological cell can be entrapped in a polyelectrolyte matrix in the form of a gel. This approach was reported by Schenkmayerová et al. [105]. An oxygen electrode, modified with a gel consisting of biomass (containing a whole cell) and natural polyelectrolytes, was used for the amperometric monitoring of (±)-cis-bicyclo [3.2.0]hept-2-en-6-one (substrate of Baeyer–Villiger oxidation).

### 3.2. Sensing Films Consisting of Three or More Components

Besides the incorporation of a sensing or mediating component into the sensing layer, casting/dropping methods are suitable for the preparation of more complex “cocktails” that include other ingredient(s). Usually, the other constituents are carbonaceous nanomaterials, as they can improve the active surface area and increase electron transfer towards the electrode surface. In Table 6, various polyelectrolyte-based sensing layers composed of more than one constituent are presented.

#### 3.2.1. Sensing Film Comprising Me or MeO NPs

A composite consisting of a carbon material, metal nanoparticles, and polyelectrolytes can be obtained by the simple preparation of PtNPs in the presence of PDDA and GO [106]. The reduction, with BH_4_^−^, of the mixture of PtCl_6_^−^, GO, and PDDA results in a composite of reduced GO (rGO; in manuscript referred to as graphene sheets), PtNPs, and PDDA. This composite was dispersed in water and used for the modification of a GCE in order to determine gallic acid by voltammetry. 

Similarly, a composite consisting of PSS, PdNPs, and graphene (rGO; referred to as graphene in manuscript) was obtained. The water dispersion of the prepared composite was deposited onto a GCE by drop-casting in order to obtain a sensor for amaranth (azo-colorant) [107]. 

During preparation, different reduction procedures were used to obtain AuNPs, PEI, and MWCNT-based composites [108]. The authors employed the thermal reduction of a mixture consisting of PEI, MWCNTs, and AuCl_4_^−^ (by heating the composite mixture to 70 °C) to obtain AuNPs. The prepared composite was dispersed and placed onto an Au electrode by drop-casting in order to develop a voltammetric sensor for dopamine. 

Li et al. [109] showed that aminophenol isomers can be easily detected by differential pulse voltammetry using a GCE decorated with AuNPs and an rGO composite coated with PDDA. The composite was prepared by the sequential thermal reductions of GO and AuCl_4_^−^ in the presence of PDDA. PDDA role was not only to act as a reducing agent but also as an electron-transport “bridge” (scaffolding layer) and a stabilizing agent for AuNPs. 

Polydiallyldimethylammonium-chloride (PDDMAC) is used as an agent for the complexation of AuCl_4_^−^, as well as an electrolyte during the electroreduction of gold nanoparticles (AuNPs) onto platinum, stainless-steel, or ITO electrodes [110]. The obtained AuNPs were stripped from electrodes by ultrasonication and complexed with carbon ink and Nafion^®^. Such a prepared composite was drop-cast onto a GCE electrode. This structure was applied to the electrochemical determination of dopamine. 

#### 3.2.2. Sensing Film Comprising DNA 

Porfir’eva et al. [111] proposed poly-aminated thiacalix [4], arene shielded DNA, and PSS or a PAA-based film to increase the sensitivity of doxorubicin. Thiacalix [4] arene acts as a protector of the negative charge of phosphate groups. This composite was applied onto a GCE that was previously coated with a redox mediator (Neutral Red or PANI). The determination principle was based on the intercalation properties of the analyte (doxorubicin) that was observed through impedimetric measurements.

#### 3.2.3. Sensing Film Comprising an Enzyme

Glucose oxidase, previously dispersed into graphene oxide, was immobilized with a cross-linked ferrocene-modified polyelectrolyte (e.g., PAA, PEI, and PAM) in order to obtain a sensing composite for the modification of a GCE surface. This composite consisted of GO, MWCNTs, PE, and an enzyme. The obtained architecture was used as selective glucose sensor [112].

The encapsulation of GOx into the chitosan–carrageenan polyelectrolyte complex and further modification with AuNPs to obtain a composite applicable for drop-casting onto a gold electrode was reported by Rassas et al. [113]. The authors detected glucose by square-wave voltammetry (SWV) using the developed sensor.

A biocompatible rGO-based composite, prepared through the electrostatic assembly of rGO with PDDA, was used as a host matrix for microperoxidase-11 (MP11) [114]. Before the enzyme immobilization, AuNPs protected with a bilayer of dimyristoyl-sn-glycero-3-phosphatidil glycerol (DMPG) were mixed with a rGO@PDDA composite. An enzyme electrode was fabricated by dropping a suspension onto a GCE. The prepared sensor was applied to the electrochemical detection of H_2_O_2_ released from living cells.

GOx immobilization on a metal (Au or magnetic) nanoparticle surface endeavors to achieve a higher surface density of the immobilized enzyme due to a high surface-to-volume ratio, was reported by Nouira et al. [115]. MeNPs coated with PAH were used as a substrate for the immobilization of the GOx in the presence of BSA and glycerol. The prepared enzyme-modified AuNPs were dispersed in a phosphate buffer and applied, by drop-casting, onto planar interdigitated electrodes (IDEs) after cross-linking by exposing the sensor to glutaraldehyde vapors, was performed. The sensor was used for the determination of glucose.

**Figure 12:**
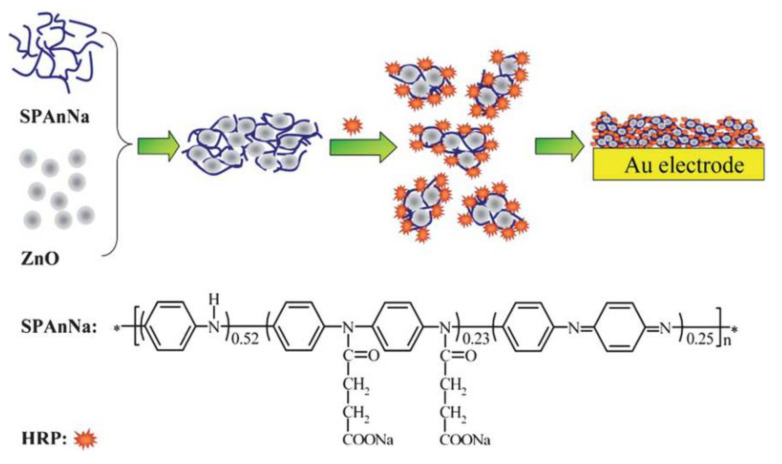
Schematic representations of preparation of an HRP−SPAnNa−ZnO/Au electrode. Republished with permission of the Royal Society of Chemistry from [116].

The polyelectrolyte properties of the conductive polymer poly(aniline-co-sodium N-(1-one-butyricacid) aniline) were of use when preparing a nanocomposite with ZnO and HRP through the simple mixing of water solutions of the respective compounds [116]. The prepared mixture was dropped onto a gold electrode (Figure 12). The developed sensor was used for the amperometric determination of H_2_O_2_.

Conjugated (conductive) polymers, with charged side groups, were used as polyelectrolytes (they can be referred to as conjugated polyelectrolytes) in the preparation of composites consisting of SWCNTs and GOx [117]. Prepared solutions were used for the coating of a GCE electrode in order to determine glucose by amperometry.

The ultrasensing determination of paraoxon was performed with SPCE modified with a composite consisting of rGO, AuNPs, and AChE [118]. A simultaneous reduction of GO and AuCl_4_^−^ was performed by BH_4_^−^ in the presence of PDDA. The obtained composite was cast onto an SPCE. PDDA was found to stabilize AChE while also being characterized by a high loading capacity and improved activity.

**Figure 13:**
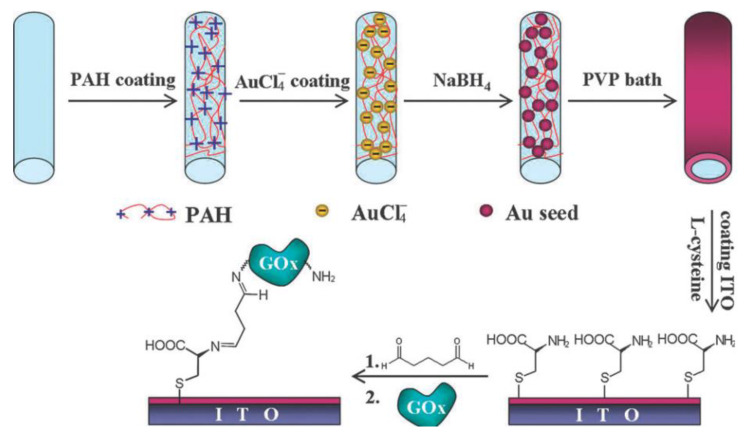
Schematic diagram illustrates formation of SiO_2_@Au core-shell fibers and the immobilization of GOx. Republished with permission of the Royal Society of Chemistry from [119].

SiO_2_ nanofibers (NFs) were introduced as a substrate (obtained by electrospinning) for the adsorption of AuCl_4_^−^, followed by its reduction with BH_4_^−^ [119]. The phosphate dispersion of functionalized SiO_2_NFs@AuNPs was introduced into a solution of L-cysteine, glutaric dialdehyde, and GOx. The obtained mixture was dripped onto an ITO electrode to obtain an electrochemical sensor for glucose (Figure 13).

The electrochemistry of proteins, with the heme prosthetic group, in complexes with weakly biocompatible polyelectrolyte copolymers (PHEMA-b-PDMAEMA) was studied in order to determine H_2_O_2_ [120]. PHEMA-b-PDMAEMA ensured a biomembrane-like film that was a favorable micro-environment for proteins. For the improvement of the electrical conductivity of the biomembrane film, MWCNTs were also incorporated. A simple mixture, consisting of PHEMA-b-PDMAEMA, MWCNTs, and hemoglobin, was cast onto a GCE and was utilized in the amperometric determination of H_2_O_2_.

### 3.3. PE in Preparation of Scaffolding Layer by Drop-Casting

The properties of polyelectrolytes can be used for the immobilization of various materials, including a redox mediator and/or an electron transfer mediator (e.g., carbon-based nanomaterials) for the preparation of a scaffolding layer (or a mediating film) applicable for further modification with a sensing layer. Additionally, a polyelectrolyte and PECs can fulfil these requirements. In Table 7, a sensor architecture with a scaffolding layer based on a polyelectrolyte containing various species is presented.

#### 3.3.1. Redox Mediators (Complex Compounds)–PE Composite

The electrosynthesis of Prussian blue (PB) at graphite and an ITO electrode surfaces modified with a PAA-SDS layer was performed in order to obtain a PB-modified electrode capable of acting as a sensor for the detection of H_2_O_2_ [121]. PAA acts as a scaffolding layer for the incorporation of PB, while the surfactant retains the tendency of the polyelectrolyte to be assembled in the form of a layered structure.

An osmium-modified PAA, soluble in dimethylformamid DMF (in presence of surfactant), was used for the modification of graphite or gold electrodes to obtain a stable and redox mediating film that is applicable for the adsorption of GOx [122]. A polypyridyl osmium complex was covalently attached to PAA and acted as a redox mediator.

#### 3.3.2. Me or MeO NPs–PE Composite

The cross-linking (e.g., stabilization) of metal NPs by SiPy^+^Cl^–^ in order to obtain a substrate for the immobilization of thiolactic acid (TLA) was reported by Mossanha et al. [123]. A suspension of AuNPs stabilized with PDDA was cast onto a GCE, after which TLA was immobilized. TLA was required for the covalent immobilization of HRP via an EDC/NHS reaction (carbodiimide reaction). The prepared sensor was successively exploited for the voltammetric determination of catechol.

#### 3.3.3. Carbon-Based Nanomaterials–PE Composite

The drop-casting of a Nafion^®^ composite with MWCNTs as an “interlayer,” in order to improve electron transfer between the sensing layer (consisting of the Nafion^®^-metal porphyrins) and a GCE, was applied in the development of an amperometric sensor for the detection of H_2_O_2_ [124].

#### 3.3.4. Me or MeO NPs–Carbon-Based Nanomaterials–PE Composite

A dual-enzyme biosensor made for the continuous and simultaneous monitoring of glucose and L-lactate was presented by Yu et al. [125]. A dual glassy carbon electrode was firstly modified with a carbon mesoporous material modified with PDDA and PtNPs by drop-casting; afterwards, the drop-casting of GOx or LOD entrapped with PEI was applied too different part of the GCE. For the additional stabilization of the enzyme layers, cross-linking by glutaraldehyde was performed.

PDDA-functionalized rGO was used in the preparation of rGO modified with AgNPs [126]. The suspension of the prepared composite was used for the decoration of a GCE. On such prepared surface, the solution of an aptamer was cast. The developed sensor was used to entrap chloramphenicol and for its determination by linear sweep voltammetry. The sequential drop-casting of a variety of polyelectrolyte composites in order to overcome problems related to electron transfer, sensitivity, etc., was also reported.

Except for having the role of a dispersant, PDDA, as a positively charged polyelectrolyte, can act as “glue” between rGO and PtCl_6_^−^, resulting in PtNPs that are synthesized in situ at the surface of rGO [127]. The mechanism is based on the exchange of Cl^−^ as a doping anion in PDDA with PtCl_6_^−^. This results in the formation of “nanoreactors” (molecular reactors) due to the presence of a number of exchangeable Cl^−^ within the long chains of PDDA. Microwave-assisted heating can be performed in order to reduce Pt(VI). This reduction method provides a microenvironment for obtaining a hybrid with a controllable density, a uniform distribution, and a small size. Various sensing layers can be applied onto this scaffolding layer. The authors chose HRP as a model enzyme and successfully tested the obtained sensor for the detection of H_2_O_2_.

A similar principle was applied in work of Hossain et al. [128] with a difference in the reduction path of PtCl_6_^−^. The authors arranged the surface of a SiO_2_/Ti and Au electrode with a composite of made of PDDA and rGO; afterwards the chemical reduction of the adsorbed PtCl_6_^−^, with ascorbic acid, was performed. The prepared electrode was applied in the amperometric determination of ascorbic acid and dopamine.

A chitosan–graphene suspension was placed onto a GCE as a scaffolding layer for the adsorption of AuCl_4_^−^, followed by the electrochemical reduction of Au(III) by cyclic voltammetry [129]. On such a prepared layer, an antibody (selective for prolactin) was immobilized in order to obtain a sensing layer applicable in the voltammetric determination of prolactin using the sandwich method.

A label-free electrochemical sensor for the voltammetric determination of angiogenin, based on immobilization of an aptamer onto AuNP-coated graphene nanosheets, was reported by Chen et al. [130]. Firstly, the composite consisting of AuNPs, PDDA, and exfoliated graphene sheets was prepared by mixing PDDA and exfoliated graphene with AuCl_4_^−^, followed by the chemical reduction of AuCl_4_^−^ with citrate. The obtained composite was cast onto a GCE, after which the immobilization of an aptamer was achieved by drop-casting. Its analytical signal was based on an increase in the electron transfer resistances in the presence of a ferri/ferro redox couple due to hybridization. This behavior manifested in the form of voltammetric current decrease.

In order to improve the immobilization of dsDNA on the pencil-graphite electrode surface, a mixture of nanomaterials (MWCNTs and TiO_2_NPs) and PDDA or chitosan was immobilized at the electrode surface [131]. The presence of this layer was found to dramatically improve the immobilization of the dsDNA by covalent and electrostatic immobilization. Such immobilized dsDNA was a subject of voltammetric oxidation experiments.

#### 3.3.5. PE or PEC as a Scaffolding Layer

The detection of dissolved oxygen at the electrode prepared by the electrodeposition of an indigo tetrasulphonate (ITS) film onto a previously modified GCE electrode with cross-linked (via gluthaldehyde) poly-L-lysine was reported by Tsai et al. [132]. The authors used this electrode in the rotation mode for voltammetric measurements.

A rapid method for the amperometric detection of anionic surfactant-CTAB, on an electrode modified with acridine orange (AO), was proposed by Hao et al. [133]. As support for the immobilization of AO, a layer consisting of PEI and PSS was obtained through the successive dip-coating of GCE in solutions of PEI and PSS.

#### 3.3.6. Other Uses of PE in Casting/Coating Mode

All-MWNTs films for the amperometric detection of NADH was presented by Sun et al. [134]. The authors made the surface of a GCE positively charged by the immersion of the electrode into PAA. The latter also provided an introduction of amine groups onto the GCE surface. Such a modified surface was exposed to active carbodiimide ester-functionalized MWCNTs. These MWCNTs were prepared by the activation of carboxylated MWCNTs (CMWNTs) by EDC and NHS to convert carboxyl groups into active carbodiimide esters. Afterwards, the prepared surface was exposed to the aminated MWCNTs (AMWCNTs). This was done in order to form amide bonds between activated CMWCNTs and AMWCNTs. The latter two steps were performed using the LbL method. The prepared all-MWNTs film had an advantage over films containing polyelectrolytes as binders for carbon nanomaterials in the context of a better sensitivity and a higher selectivity. In addition, the reported structure exhibited an excellent stability due to covalent interlayer bonding.

L,L-diphenylalanine micro/nanostructures (FF-MNSs), as enzyme support for the development of a sensing platform for the determination of glucose, was introduced by Kogikoski et al. [135]. FF-MNSs modified with microperoxidase (MP-11) acted as protein-like structures and redox mediators due to a heme-containing group on MP-11. The orthorhombic structure of the scaffolding layer (11-FFMNSs-MP11) was *p*-type doped by PAH, thus resulting in a smaller charge-transfer resistance. MP-11 in the orthorhombic FF-MNSs showed a better sensitivity due to shifts in the orbital energies of the Fe atom within the heme group. This made it more reactive when it was outside of the porphyrin ring plane, thus allowing for the oxidation of H_2_O_2_ to be more effective. This composite was a scaffolding layer to cast GOx and to further modify the prepared layer with Nafion^®^.

### 3.4. PE or PEC as Sensing Layer

In addition to their “auxiliary” function in the sensing layer (as matrix, substrate for immobilization, etc.), polyelectrolytes may have roles as sensing elements or in the improvement of the preconcentration of an analyte and in increasing selectivity. As the polyelectrolyte layer ensures a high electroactive area and can effectively enhance the accumulation of an analyte near the electrode surface due to electrostatic interactions between the analyte and the polyelectrolyte; this can obviously improve the preconcentration of the analyte and the sensitivity of the electrode. Additionally, polyelectrolyte films can reduce or even eliminate the influence of interferences (of the same charge) by simple electrostatic repulsion, thus improving selectivity. The application of PE or PEC as sensing layers in various electrode architectures is presented in Table 8.

Natural polyelectrolytes, chitosan, and pectin were used in the preparation of PEC [136]. The prepared water dispersion of PEC was applied onto a GCE by drop-casting and used for the determination of an antibiotic drug and a herbicide—metronidazole and metribuzin, respectively. Enhanced electrocatalytic activity, compared to a bare GCE, was attributed to the stochiometric combination of polyelectrolytes that can lead to enhanced electrocatalytic activity; the electrostatic interaction of nitro, imine, and carboxyl groups present in analytes and a protonated amino group in chitosan; hydrogen bonding between PEC and analytes; a large surface area; and swelling properties that could provoke absorption of analytes.

Additionally, chitosan can be used for enantioselective drug recognition due to the chiral properties of its surface. Its instability and solubility in water prohibits it from being used as a sensing element. However, to improve the physical properties of a sensing layer, chitosan can be complexed with various species. Zilber et al. [137] used sulphonated chitosan and cyclodextrin to prepare PEC, appropriate for the modification of a GCE. The prepared electrode was applied to the voltammetric determination of atenolol enantiomers.

A GCE covered with a PDMDAAC-containing film was successfully utilized for the improvement of analytical signal in the voltammetric determination of nitrite [138]. A PDMDAAC-containing film, obtained by drop-casting, facilitated the preconcentration of an analyte and enhanced its selectivity. The sensitivity of PDMDAAC was improved through the formation of a PEC containing cellulose sulphate.

Screen-printed graphite electrodes, modified by the drop-casting of a polymeric matrix based on PAA and a surfactant (SDS), were utilized for the impedimetric determination of ethyl xanthan [139]. The method was based on the measurement of charge-transfer resistance. Applying anodic potential before impedance measurements triggered the oxidation of xanthan to dixanthogen, preventing the electron exchange of the redox probe (ferri/ferro cyanide system) at the working electrode, resulting in an increase in the charge-transfer resistance.

The electrostatic interaction between PAH and NADH was used for the accumulation of NADH at the surface of a modified electrode; afterwards the oxidation of NADH was performed with differential pulse voltammetry [140]. An SPCE was used as a working electrode.

Additionally, the presence of a polyelectrolyte in a sample solution during electrochemical determination can provide an increase in the concentration of an analyte near the electrode surface via electrostatic attractions. The knowledge of this principle was used by Prakash et al. [141] in order to determine uric and ascorbic acid using a GCE.

A gas sensor for formaldehyde, based on SPCE modified with hydrazinium polyacrylate (HPA), was reported by Menart et al. [142]. HPA, with its accumulation capabilities and possibility of derivatization (based on hydrazine-involved reaction), can be applied in the voltammetric determination of formaldehyde. The authors also applied PAA, for the same purpose, though without any success.

A carboxylated poly(N-(1-one-butyric acid)benzimidazole) (PBI-BA) was synthesized in order to develop H_2_O_2_ sensors [143]. This principle of an enzyme-free H_2_O_2_ sensor was based on the oxidation of imines (present in the backbone of a polyelectrolyte) by percarboxylic acid (formed in a reaction between the carboxylic groups present at side-chains of PBI-BA and H_2_O_2_). The analytical signal arose from the electrochemical reduction of imine oxidation products. In fact, the authors used a composite made of rGO (in order to provide superior conductivity and an increased surface area) and a polyelectrolyte for the modification of a gold electrode.

A simple impedance sensor, based on PAH, was reported by Bronder et al. [144]. The polyelectrolyte layer of PAH, placed on a silica chip, was used for the detection of adsorbed dsDNA molecules by monitoring the surface-potential changes induced by intrinsic molecular charge.

### 3.5. Other Methods Involved in Application of PEs in Sensor Architectures

#### 3.5.1. Electrospinning and Spin-Coating of PE onto a Working Electrode

PEs can also be cast onto a substrate surface of by electrospinning in order to obtain a suitable support with a large specific area for the immobilization of various sensing materials. Additionally, the obtained PEs electrospun/nanospun fibers can improve selectivity of a sensor since their structure acts as a size-selective membrane. This approach for electrochemical sensing system development is presented in Table 9.

A composite of crosslinked and quaternized cationic polyelectrolyte QC-P4VP (poly(4-vinyl pyridine)) nanofibers coated with AgNPs was explored for the development of impedimetric humidity/gas sensors [145]. P4VP was placed onto a gold electrode by electrospinning, after which quarterization with 1,4-dibromobutane (DBB) was performed (obtained product: QC-P4VP). The authors found that the electrospinning condition greatly affected the morphology and sensing properties of nanofibers. The prepared nanofibers were decorated with AgNPs in order to decrease impedance and to affect the hydrophilicity of the prepared composite.

The electrospun nanofibers of polyamide and PAH (probably in order to enhance the rate of MWCNTs adsorption) was placed onto an ITO electrode; afterwards, coating with MWCNTs was performed [146]. The prepared electrode was used in the voltammetric determination of dopamine.

**Figure 14:**
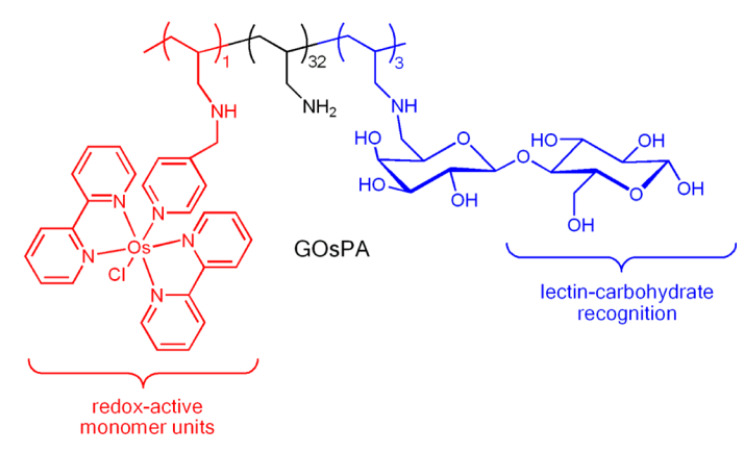
Redox-active glycopolyelectrolyte (GOsPA). Reprinted with permission from [147]. Copyright (2013) American Chemical Society.

An alternative approach to the LbL construction method was proposed by Lorena Cortez et al. [147]. A self-assembled supramolecular architecture consisting of a redox-active Os(bpy)_2_-containing glycopolyelectrolyte-surfactant (SDS) lamellar assembly and protein building blocks (based on Concanavalin A) was developed in order to immobilize HRP (Figure 14). Concanavalin A is a lectin. It is well-known that lectins can generally act as bio-affinity bridges between a sugar (glycopolyelectrolyte) and a protein (enzyme) through electrostatic attractions. A gold electrode was modified with a glycopolyelectrolyte—surfactant lamellar assembly by spin-coating, followed by the immersion of such a modified electrode into Concanavalin A (or into Concanavalin A modified with an osmium complex). Afterwards, the modified electrode was immersed into an HRP solution. This electrode was utilized for the amperometric determination of H_2_O_2_.

Silica sol, prepared by the hydrolysis of tetraethyl orthosilicate (TEOS), was modified in order to obtain a cationic (with film of the PDDA and PVS) or an anionic (with PDDA) ion-exchange material [148]. After the addition of adenine, the prepared material was placed onto a GCE or a glass substrate by spin-coating. Adenine (uncharged at neutral pH) was rinsed from the prepared film with a mixture of ethanol and water. The developed senor was applied to the voltammetric determination of adenine and guanine.

#### 3.5.2. PEs in Construction of Reporter Probes, Nanoprobes, etc.

PEs have the ability to act as stabilizing agents in the preparation of various kinds of nanoparticles, and they can also change a charge of prepared nanoparticles; they are often used in the preparation and functionalization of nanoparticles. These functionalized nanomaterials can be used as amplification markers in certain types of electrochemical sensors (e.g., reporter probes and nanoprobes in sandwich or competitive methods of analysis). In Table 9, the application of PEs in this context is presented.

An HRP-labelled DNA probe immobilized onto CNTs functionalized with PDDA, in order to achieve a positive surface charge needed for adsorption of PtNPs, was reported by Dong et al. [149]. The authors used this probe in a sandwich-type DNA sensor in order to catalyze (after hybridization) the oxidation of a substrate (3, 3′, 5, 5′ tetramethylbenzidine (TMB)) using H_2_O_2_. An amperometric signal originates from reduction of TMB.

An immunosensor for human immunoglobulin G, based on a coated mesoporous carbon nanosphere as a nanoprobe, was reported by Lai et al. [150]. A mesoporous carbon nanosphere was decorated with Prussian blue; afterwards, coating with PDDA was performed. Such a decorated nanosphere was further modified with AuNPs (via the reduction of AuCl_4_^−^ with citrate). Furthermore, the modification was performed through the immobilization of a labelled (GOx) probe antibody through the inherent interaction with AuNPs. The prepared nanoprobe was used in the determination of human immunoglobulin G by voltammetry using a sandwich type method in the presence of glucose as a substrate.

An electrochemical immunosensor for the ultrasensing detection of oral cancer biomarker interleukin-6 (IL-6) was reported by Li et al. [151]. The authors used ferrocene (FC)-coated polyelectrolyte nanoparticles (FC-PPN) as a label. The polyelectrolyte nanoparticles were prepared by the adsorption of PDDA and PSS onto a CaCO_3_ nanoparticle surface; simultaneously, FC was encapsulated into a polyelectrolyte layer. A secondary antibody (for IL-6) was immobilized onto the surface of FC-PPN via electrostatic attractions. The sandwich method was used for the detection of IL-6 on a GCE electrode modified with GO coated with a primary antibody through the amidation of -COOH of the GO and -NH_2_ present in the antibody. In this case, the authors used voltammetry to obtain an analytical signal.

The immobilization of a gene-sensing material, produced by the electro-copolymerization of ThPhEG and modified oligonucleotide (ON) ThPhCONH-ON onto PAA brushes, was reported by Kerr-Phillips et al. [152]. The brushes were produced by grafting tert-butyl acrylate (tBA) together with surface-initiated ARGET ATRP (activators regenerated by electron transfer atom transfer radical polymerization) on electrospun PEDOT-embedded rubber. After hybridization in solution, EIS experiments were performed using ferri/ferro redox couple. The change in the charge-transfer resistance, compared to the resistance before incubation, was taken as a signal for hybridization.

## 4. Conclusions and Future Perspectives

Though many comprehensive reviews concerning the application of polyelectrolytes in various sensing architectures have been highly informative; they have mostly been related to certain analytes only, used materials, or applied methods/procedures for obtaining sensing layers. In contrast, this review is focused on sensing layer architectures and the role of polyelectrolytes in such prepared sensing layers. Furthermore, the applicability and the role of polyelectrolytes in the preparation of various marked nanomaterials, for competitive or sandwich determination methods, is also presented.

Some standard features of polyelectrolytes, such as their ability to act as interlayers between a sensing/mediating/transducing layer and an encapsulating agent for various inorganic species, biomolecules, or nanoparticles in sensing/mediating/transducing layers, can be obtained using the LbL method. Sensing layers obtained by the LbL method have different architectures. They can be produced in form of alternating bilayers, layers consisting of more than two layers, or layers consisting of two or more alternating bilayers. Additionally, the LbL method, involving polyelectrolytes, was used in the preparation of a scaffolding layer in order to facilitate the immobilization of various sensing/mediating/transducing elements through casting/coating methods or covalent immobilization. Additionally, this multilayer can minimize the interference of a substrate or provide a uniformly charged surface. Furthermore, the functional integration of nucleic acids or proteins and other nanoparticulate materials (as synthetic polymer-based materials) can be achieved via polyelectrolyte multilayer films (or modified versions of films with metal or metal oxide nanoparticles) deposited onto the surface of a nanoparticulate material. These materials can provide a predefined and biocompatible cargo-carrying and targeting capability that can be used in the preparation of the sensing layer at a solid-state electrode surface or in the preparation of competitive particles or labelled particles for the competitive or the sandwich method, respectively.

Moreover, polyelectrolytes are often used in casting/coating methods for the modification of electrode surfaces due to their capability to entrap various species with sensing/mediating/transducing properties. The synergistic effects of these materials can significantly improve the analytical properties of sensors. Hence, this could lead to the development of various sensing composite materials. In addition, PEC can be used as sensitive layers themselves.

Other specific properties of polyelectrolytes revealed in the presented articles are related to the enhancement of electron transfer or a conductive bridge between a sensitive layer and an electrode substrate due to presence of aromatic rings in PE, improved adhesion properties between nanoparticles and an electrode substrate, the stabilization of an enzyme and a nucleic acid together with high loading and improved activity, their role as a glue between mediating materials (e.g., redox mediator) and a transducer (e.g., carbon nanomaterials), the introduction of some chemically reactive groups (amino) to an electrode surface with a potential for further covalent or electrostatic bonding, the reduction properties (PDDA) in the preparation of metal NPs by chemical reduction, the elimination of interferences (of the same charge) by electrostatic repulsion, and the increase of accumulation of an analyte near or on an electrode surface due to electrostatic interactions, thus improving the sensitivity of sensors.

Therefore, it is obvious that significant efforts and different procedures have been made in order to produce optimal sensing layers based on polyelectrolytes. However, the control of the assembly and the possibility of tuning the composition, structure, and dimension of the prepared sensitive layers remain a challenge. The significant potential of the polyelectrolytes offers many opportunities for taking one step toward improving future burgeoning developments in the field of electrochemical sensing. Firstly, this means stepping away from the standard manual procedures of multilayer production. Compared to conventional dispensing, the electrohydrodynamic (EHD) printing process seems to be a promising strategy for obtaining various sensitive multilayers with a tunable composition, morphology, and electrical properties, thus leading to controllable sensing functions. In addition, this technique is not restricted to the incorporation of certain nanomaterials because it can be used to prepare layers that contain nanoparticles of different dimensionalities: zero-dimensional (0D, e.g., incorporating NPs), one-dimensional (1D, e.g. incorporating CNTs), two-dimensional (2D, e.g., incorporating rGO), and three-dimensional (3D, e.g., incorporating nanoshells). Secondly, a tremendous progress can be expected in the development of stimuli-responsive hydrogel materials based on polyelectrolytes for sensing purposes. This structural platform can be considered in the construction of self-healing hydrogel sensors with promising applicability for “in situ” or “in vivo” sensing. More effort has to be made in the process of the printing of these materials, as only few varieties are suitable for printing (so called 4D printing, which is, in fact, the 3D printing of “smart” materials). Additionally, electroactive polymers could be the subjects of 4D printing. Thirdly, some improvements can be expected in the application of polyelectrolytes in the fabrication of sensitive layers on transparent conducting films (TCFs). This sensor architecture may be particularly useful in solving problems related to mechanical strength, adhesion, stretchability, etc. Moreover, TCFs offer unlimited possibilities for the development of wearable sensors (e.g., on skin and organs) to monitor physiological processes. Finally, a detailed overview is given of these interesting topics in comprehensive review articles [153,154,155,156].

## Figures and Tables

**Table 1:** Summary list of electrochemical sensors based on sensing multilayer films (prepared by the LbL method) consisting of two components that act as self-containing layers.

2.1. Multilayer Film Consisting of Two Components (Layers)								
2.1.1. PE and Sensing/Mediating/Transducing Component								
Redox Mediators (Complex Compounds) as a Layer								
PE(s) Used	Sensing Element	Modification Methods	Electrode/Substrate	Path Of Modification	Sensor Construction	Analyte	Analysis Method	Reference
SiPy(+)Cl(−)	Tetra sulpho phthalocyanine NiTsPc	LbL	Indium tin oxide (ITO)	LbL: SiPy(+)-NiTsPc(−)	ITO/LbL: SiPy-NiTsPc	Dopamine	Square-wave voltamm-etry (SWV)	[32]
Poly(diallyldimethylammonium chloride) (PDDA(+))	Copper tetra-sulphonated phthalocyanine CuPcTS	Drop-casting/LbL	Glassy carbon electrode (GCE)	Drop-casting of the CNT(−); then LbL: PDDA(+)-CuPcTS(−)	GCE/carbon nanotube (CNT)/LbL: PDDA-CuPcTS	2-mercaptoethanol	Ampero-metry (AMP)	[33]
Poly(ethylenimine) (PEI(+))	Phosphotungstic acid-PTA H_3_PW_12_ O_40_	LbL	ITO	LbL: PEI(+)-PTA(^−^)	ITO/LbL: PEI-PTA	Ascorbic acid and H_2_O_2_	Cyclic voltamm-etry (CV)	[34]
Poly(styrene sulphonate) (PSS(−)); Nafion^®^(−)	Fe(III)porphyrine Fe(III)Ph	Drop-casting/LbL	Screen-printed carbon electrode (SPCE)	Suspension of multi-walled carbon nanotube (MWCNT)(−)@Nafion^®^(−) onto SPCE; then LbL: PSS(−)-Fe(III)P(+)	SPCE/MWCNT@Nafion^®^/LbL: PSS-Fe(III)P	Bromate	CV	[35]
Poly(allylamine hydrochloride) (PAH(+)); PSS(−); surfactant Triton-X^®^	Tungstophosphate (POM)	Silanization of GCE/immersion/LbL	GCE	Silanizated GCE by treatment with Aminopropyltriethoxysilane—ATS(+); then immersion in PSS(−); than LbL: PAH(+)@Trinton-X^®^-POM(−)	GCE@ATS/PSS/LbL: PAH@Trinton-X^®^-POM	Nitrite	AMP	[36]
**Me or MeO NPs as a Layer**								
PDDA(+)	IrO_x_NPs (nanoparticles)	LbL	ITO	LbL by printing: PDDA(+)-IrO_x_NPs(−)	ITO/LbL: PDDA-IrO_x_NPs	pH	Potentio-metry (POT)	[37]
Polyviologen (PV(+))	AuNPs	LbL	Au	Au modified with 2-mercaptoethansulphonate (MESA)(−); then LbL: PV(+)-AuNPs(−)	Au@MESA/LbL: PV-AuNPs	H_2_O_2_	AMP	[38]
Poly ((2-ethyldimethylammonioethyl methacrylate ethyl sulphate)-co-(1-vinylpyrrolidone) (PQ11(+))	PQ11-AgNPs	LbL	ITO	LbL: PQ11(+)-AgNPs	GCE/LbL: PQ11-AgNPs	H_2_O_2_	AMP	[39]
**Carbon-Based Nanoparticles as a Layer**								
PDDA(+)	MWCNTs	LbL	GCE	LbL: PDDA(+)-MWCNT(−)	GCE/LbL: PDDA-MWCNT	Caffeine	Different-ial pulse voltamm-etry (DPV)	[40]
PAH(+)	Graphene oxide (GO)	LbL	GCE	LbL: PAH(+)-GO; then reduction of GO with NaBH_4_ to obtain rGO.	GCE/LbL: PAH-rGO	Cu(II)	Different-ial pulse anodic stripping voltamm-etry (DPASV)	[41]
conjugated PE: PBCSO_3_(−)	Reduced graphene oxide (rGO)	Electrochem. deposition/LbL	GCE	Electrochemical deposition and simultaneous reduction of graphene oxide (GO) on GCE; then LbL: PBCSO_3_(−)-rGO	GCE/ErGO/LbL: PBCSO_3_-rGO	Polycyclic AromaticHydrocarbons	DPV	[42]
**DNA as a Layer**								
PAH(+)	Single stranded DNA (ssDNA)	LbL	SiO_2_	SiO_2_(−) at physiological pH; then LbL: poly(acrylamide) (PAH(+))-ssDNA(−)	SiO_2_/LbL: PAH-ssDNA	cDNA	POT	[43]
PAH(+)	ssDNA	LbL	SiO_2_	SiO_2_(−) at physiological pH; then LbL: PAH(+)-ssDNA(−)	SiO_2_/LbL: PAH-ssDNA	cDNA	Impedimetry (IMP)	[44]
PAH(+)	ssDNA	LbL	FE chip SiO_2_	LbL: PAH(+)-ssDNA(−)	FE chip/LbL: PAH-ssDNA	cDNA	Electrochemic-al imped-ance spectro-scopy (EIS)	[45]
PAH(+)	ssDNA	LbL	FE chip SiO_2_	LbL: PAH(+)-ssDNA(−)	FE chip/LbL: PAH-ssDNA	cDNA	IMP	[46]
PAH(+); PSS(−)	DNA	Electropolymerization/LbL	GCE	Electropolymerization of Methylene green (MG) or Methylene blue (MB) onto GCE; then different architectures were applied using the LbL method:: 1. PSS-PAH; 2. DNA-PAH; 3. PSS-PAH-PSS; 4. PSS-PAH-DNA; 5. DNA-PAH-PSS.	GCE/MG or MB/LbL: DNA-PAH	DNA	IMP	[47]
**Enzyme as a Layer**								
PDDA(+); PAS(−)	Choline oxidase (ChOx)	Drop-casting/LbL	SPCE	1. Drop-casting of the sol MnO_2_; then drop-casting of PDDA(+); then drop-casting of ChOx 2. Drop-casting of the sol solution of MnO_2_; then LbL: PDDA(+)-PAS(−); then drop-casting of ChOx 3. Drop-casting of the sol solution of MnO_2_; then LbL: PDDA(+)-ChOx	1. SPCE/MnO_2_/PDDA/ChOx 2. SPCE/MnO_2_/LbL:PDDA-PAS/ChOx 3. SPCE/MnO_2_/LbL: PDDA-ChOx	Choline	AMP	[48]
PEI(+)	Urease	LbL	Field-effect transistor (FET) (Si-SiO_2_)	glass substrate functionalized with APTES; then drop-casting of GO followed by its chemical reduction; then functionalization by SPS(−); then LbL: PEI(+)-urease(−)	FET@APTES/rGO/SPS/LbL: PEI-urease	Urea	AMP	[49]
PAH(+)	Sarcosine oxidase (SOD)	LbL	Au	Benzenedithiol (BDT) was immobilized onto Au; then immersion of the Au electrode into Cd/Se quantum dots (Cd/SeQD) to obtain Cd/SeQD@BDT; then LbL: SOD-PAH(+)	Au@BDT/Cd/SeQD@BDT/LbL: SOD-PAH	Sarcosine	AMP-photo current	[50]
PAH(+)	GOx	LbL	Au	immobilization of the benzenethiol functionalized CdSe/ZnQDs; then LbL: GOx-PAH(+); then GTH (cross-linking)	Au/QDs/LbL: GOx-PAH	Glucose	AMP-photo current	[51]
Poly(amidoamine) dendrimer (PAMAM(+)); PAH(+)	Penicilase	LbL	FET (Si/SiO_2_)	LbL: PAMAM(+) or PAH(+)-penicilase	FET/LbL: PAMAM or PAH-penicilase	Penicillin	EIS capacitive	[52]
**2.1.2. Sensing/Mediating/Transducing Material Entrapped within PE and Used as a Self-Containing Layer**								
**Me or MeO NPs–PE as a layer**								
SiPy(+)Cl(−); HA(−)	PtNPs@SiPy	LbL	Fluorine doped tin oxide (FTO)	Reduction of the PtCl_6_^−^ in the presence of SiPy(+); then LbL: HA(^−^)-PtNPs@SiPy(+) and vice versa;	FTO/LbL: HA-PtNPs@SiP	17α-ethynylestradiol, EE2	DPV	[53]
Chitosan (CS(+)); PSS(−)	Me^n+^@CSMe^n+^ = Cu^2+^, Ni^2+^, Ag^+^	LbL	Quartz, silicon wafer; ITO	Substrate/LbL: Me^n+^@CS(+)-PSS(−); then chemical reduction of Me^n+^ in film	substrate/LbL: Me@CS-PSS	Glucose	CV	[54]
PAH(+); poly(acrylic acid) (PAA(−));	AuNPs@PAH or AuNPs@PAA	LbL	ITO	Reduction of AuCl_4_^−^ with NaBH_4_ in the presence of the PE; then LbL onto ITO: Au NPs@PAH(+)-AuNPs@PAA(−)	ITO/LbL: AuNPs@PAH-AuNPs@PAA	Nitrite	CV	[55]
poly(vinyl pyrrolidone) (PVP(0)); PAMAM(+)	CoFe_2_O_4_	LbL	Chip: Al/p-Si/SiO_2_	chips:Al/p-Si/SiO_2_; then LbL: PAMAM(+)-CoFe_2_O_4_@PVP(0)	Al/p-Si/SiO_2_/LbL:PAMAM-CoFe_2_O_4_ @PVP	H_2_O_2_	IMP	[56]
**Carbon-Based NPs–PE as a Layer**								
PSS(−); PAH(+)	rGO@PSS/rGO@PAH	LbL	carbon film	LbL: rGO@PSS(−)-rGO@PAH(+)	carbon film/LbL: rGO@PSS-rGO@PAH	NO_2_	Resistance	[57]
PDDA(+)	Enzyme-AChE	LbL	GCE	LbL: Single-wall carbon nanotubes (SWCNTs)@PDDA(+)-AChE(−)	GCE/LbL: SWCNTs@PDDA-AChE	Carbaryl pesticide	AMP	[58]

**Table 2:** Summary view of electrochemical sensors based on multilayer sensing films, obtained by LbL, consisting of more than two components.

2.2. Multilayer Film Consisting of More Than Two Components (Layers)								
2.2.1. Multilayer Film Consisting of Three or More Alternately Deposited Layers								
Redox Mediators (Complex Compounds) as a Layer								
PE(s) Used	Sensing Element	Modification Methods	Electrode/Substrate	Path of Modification	Sensor Construction	Analyte	Analysis Method	Reference
PEI(+)	NaMMT and NiTsPc	LbL	ITO	LbL: PEI(+)-MMT(−)-PEI(+)-NiTsPc	ITO/LbL: PEI-MMT-PEI-NiTsPc	Dopamine	DPV	[59]
PAH(+); PEI(+)	PB	Drop-casting/LbL	Au	Drop-casting of PEI(+) onto Au; then LbL: rGO-PAH(+)-PB(−)	Au/PEI/LbL: rGO-PAH-PB	H_2_O_2_	CV	[60]
PEI(+); PAH(+)	PB	LbL	Au	LbL: PEI(+)-PB(−)-poly(3,4-ethylenedioxythiophene) (PEDOT):PSS-PAH(+)	Au/LbL: PEI-PB-PEDOT:PSS-PAH	H_2_O_2_	CV	[61]
PEI(+); PAH(+)	PB	LbL	Au	LbL: PEI(+)-PB(−)-PPy or polyaniline (PANI)-PAH(+)	Au/LbL: PEI-PB-PPy or PANI-PAH	H_2_O_2_	CV	[62]
PAA(−); PAM(+); PDDA(+)	MoS_2_@PAA or PAM	LbL	Quartz, silicon, ITO	LbL:PDDA(+)-MoS_2_@PAA(−)-MoS_2_@PAM(+)-PEDOT:PSS(−)	substrate/LbL: PDDA-MoS_2_@PAA-MoS_2_@PAM-PEDOT:PSS	H_2_O_2_	AMP	[63]
**DNA as a Layer**								
PAH(+); PSS(−)	Methylene blue (MB) and Methylene green (MG); DNA	electropolymerization/LbL	GCE	Electropolymerization of MB or MG onto GCE; then different LbL architectures were obtained: 1. PSS-PAH; 2. DNA-PAH; 3. PSS-PAH-PSS; 4. PSS-PAH-DNA; 5. DNA-PAH-PSS.	GCE/LbL: DNA-PAH-PSS	DNA	IMP	[47]
**Enzyme as a Layer**								
PDDA(+)	LOx	LbL	Polycryst. Au (PGA)	LbL on the thiolated (MPS) PGA surface(−): PDDA(+)-AuNPs-LOx	Au@MPS/LbL: PDDA-AuNPs-LOx	L-lactate	AMP	[64]
**2.2.2. Multilayer Film Consisting of Two or More Multilayer Films**								
**Me and Meo Nps as a Layer**								
Poly(diallyldimethylammonium chloride) (PDADMAC(+)); PSS(−)	AuNPs	LbL	PDMS pattern channel	LbL: PDADMAC(+)-PSS(−); then LbL: PDADMAC(+)-AuNPs	PDMS pattern channel/LbL: PDADMAC-PSS/LbL: PDADMAC-AuNPs	-	Conductivity	[65]
PDDA(+); PSS(−)	Au	LbL	ITO	Graphene nanopellets stabilized in PDDA(+) (GPDDA(+)) and PSS(−) (GPSS(−)); LbL: GPDDA(+)-GPSS(−); then LbL: AuNP(+)-GPSS(−)	ITO/LbL: GPDDA-GPSS/LbL: AuNP-GSS	Methyl parathion	DPV	[66]
PDDA(+); PSS(−)	SiO_2_NPs	LbL	ISFET	LbL: PDDA(+)-PSS(−); then LbL: PDDA(+)-SWCNT(−) or In_2_O_3_NPs(−); then LbL: PDDA(+)-SiO_2_NPs(−)	ISFET/LbL: PDDA-PSS/LbL: PDDA-SWCNT or In_2_O_3_NPs/LbL: PDDA-SiO_2_NPs	pH	chemoresistance	[67]
**Carbon-Based Nanomaterials as a Layer**								
CS(+); PAH(+); PDDA(+); PSS(−)	CS@SWCNTs	LbL	SiO_2_ plate covered with Cr-Au	SiO_2_@Cr-Au; then LbL: PDDA(+)-PSS(−); then LbL: PE(+)-SWCNTs(−)	SiO_2_@Cr-Au/LbL: PDDA-PSS/LbL: PE-SWCNTs	pH	chemo resistance	[68]
**Enzyme as a Layer**								
PAH(+); PSS(−)	GOx	Immersion/LbL	Au	Au modified with 3-mercapto-1-propanesulphonic acid (MPS) (−); then precursor bilayer film: PAH(+)@PSS(−); then LbL: MWCNTs(−)@PAH(+)-HRP (horse radish peroxidase); then LbL: ConA (bridge between HRP and GOx)-GOx	Au@MPS/PAH@PSS/LbL: MWCNTs@PAH-HRP/LbL: ConA-GOx	Glucose	AMP	[69]
PDDA(+); PSS(−)	SWCNTs; AChE.	LbL	SiO_2_/Cr/Au	LbL: PDDA(+)-PSS(−); then LbL: PDDA(+)-SWCNTs(−); then layer of the PDDA(+); then LbL: PDDA(+)-PSS-AChE	SiO_2_/Cr/Au/LbL: PDDA-PSS/LbL: PDDA-SWCNTs/PDDA/LbL: PSS-AChE	pH, acetylcholine	POT	[70]

**Table 3:** Summary of electrochemical sensors based on the multilayer “precursor” film obtained using LbL.

2.3. Multilayer Film as a Substrate (“Precursor” Film) for Deposition of a Sensing Layer								
2.3.1. Redox Mediators (Complex Compounds) in a “Precursor” Multilayer Film								
PE(s) Used	Sensing Element	Modification Methods	Electrode/Substrate	Path of Modification	Sensor Construction	Analyte	Analysis Method	Reference
PEI(+)	Aptamers (TBA; LBA)	LbL/drop-casting	ITO	LbL: Fc@PEI(+)-CNTs(−); then drop-casting of the TBA or LBA; then bovine serum albumin (BSA) (prevent non-specific adsorption)	ITO/LbL: Fc@PEI-CNTs/TBA or LBA	Thrombin lysosome	DPV	[71]
PDDA(+); PSS(−)	Aptamer	LbL/drop-casting	ITO	LbL: MB(−)@PDDA(+)-rGO@PSS(−); then drop-casting of the aptamer; then BSA	ITO/LbL: MB@PDDA-rGO@PSS/aptamer	Chiral peptide detection	DPV	[72]
SiPy(+)Cl(−); Nafion^®^(−)	GOx	LbL/drop-casting	FTO	LbL: SiPy(+)-CuTsPc(−); then adsorption of the SiPy(+); then drop-casting of the GOx; then drop-casting of the Nafion^®^(−);	FTO/LbL: SiPy-CuTsPc/SiPy/GOx/Nafion^®^	Glucose	AMP	[73]
**2.3.2. Me or MeO NPs in a “Precursor” Multilayer Film**								
PDADMAC(+); PSS(−)	GTH	LbL/drop-casting	ITO	ITO modified with PSS(−); then LbL: MWCNTs@PDADMAC(+)-AuNP; then drop-casting of the GTH	ITO/PSS/LbL: MWCNTs@PDADMAC-AuNP/GTH	Dopamine	CV	[74]
PEI(+); PSS(−)	P_2_Mo_17_V	LbL/dip-coating	ITO	LbL: PSS(−)-PEI(+); then immersion into AuCl_4_^−^ followed by reduction with NaBH_4_; then immersion into AgNO_3_ followed by reduction with asorbic acid; then dip-coating into solution of P_2_Mo_17_V@PEI(+)	ITO/ LbL: PSS-PEI/ Au@2Ag/ P_2_Mo_17_V@PEI	L-cysteine	AMP	[75]
PSS(−); PDDA(+)	Anti-APOE-4	LbL/electro deposition	ITO	LbL: PDDA(+)-PSS(−); then potentiostatic electrodeposition from AuCl_4_^−^ to produce fractal Au (FracAu); then immobilization of anti-APOE-4	ITO/LbL: PDDA-PSS/ FracAu/anti-APOE-4	Protein APOE-4	AMP	[76]
PAH(+); PSS(−)	Au@target DNA	LbL/dip-coating	Au	immersion of the Au electrode into mercaptopropionic acid (MPA(−)); then LbL: PAH(+)-PSS(−); then chemical reduction of the AuCl_4_^−^ with NaBH_4_ onto Au; then immobilization of DNA probe	Au@MPA/ LbL: PAH-PSS/ AuNPs/DNA	Target DNA	DPV	[77]
**2.3.3. DNA in a “Precursor” Multilayer Film**								
PAA(+); PSS(−)	Enzyme AChE	LbL/drop-casting	GCE	Drop casting of the suspension of Co-phtalocyanine (CoPc) and Carbon Black (CB); then LbL: PAA(+)-PSS(−) or PAA(+)-DNA(−); then drop-casting of AChE	GCE/CoPc@CB/LbL: PAA-PSS or PAA-DNA/AChE	Huperzine A and galantamine	AMP	[78]
**2.3.4. Unmodified PEs as a “Precursor” Multilayer Film**								
PAA(+); PSS(−)	AChE	Drop casting/LbL/drop-casting	GCE	Drop-casting of suspension of the Co-phtalocyanine (CoPc) and Carbon Black (CB) onto GCE; then LbL: PAA(+)-PSS(−); then drop-casting of AChE	GCE/CoPh@CB/LbL: PAA-PSS/AChE	Donepezil and berberine	Measurement of anodic current	[79]
PDDA(+); PAS(−)	Choline oxidase (ChOx)	Drop-casting/LbL/drop-casting	SPCE	1. Drop-casting of the sol solution of MnO_2_; then drop-casting of PDDA(+); then drop-casting of ChOx 2. Drop-casting of the sol solution of MnO_2_; then LbL: PDDA(+)-PAS(−); then drop-casting of ChOx 3. Drop-casting of the sol solution of MnO_2_; then LbL: PDDA(+)-ChOx	1. SPCE/MnO_2_/PDDA(+)/ChOx 2. SPCE/MnO_2_/LbL: PDDA-PAS(^−^)/ChOx 3. SPCE/MnO_2_/LbL: PDDA-ChOx	Choline	AMP	[48]
Polysaccharide: CS(+); carboxymethylpullulan CMP(−)	Urease	LbL/ adsorption or covalent immobilization of enzyme	Carbon	PANI(^−^) electropolymerized over graphite; then LbL: CS(+)-CMP(−); then drop-cast or covalent immobilization of urease (via carbodiimide reaction)	C/PANI/LbL: CS-CMP/enzyme	Urea	POT	[80]
CMP(−); CS(+)	Urease and PEC	LbL/covalent immob. of enzyme	SPCE	Electropolymerized PANI over SPCE; then LbL: CMP(−)-CS(+); then covalent grafting via carbodiimide reaction of urease onto CMP	SPCE/LbL: CMP-CS/urease	pH and urea	POT	[81]
PDDA(+); PSS(−); Nafion^®^	GOx	LbL/dip-coating	ITO	ZnONRs or NPs deposited onto ITO; then LbL: PSS(−)-PDDA(+); then dip-coating of GOx; then Nafion^®^	ITO/ZnO NRs or NPs/ LbL: PSS-PDDA/ GOx/Nafion^®^	Glucose	AMP	[82]

**Table 4:** Summary of electrochemical sensors based on the application of the LbL method in the development of various composites.

2.4. LbL Method in Development of Various Composite Nanoparticles and Other Sensor Architectures								
2.4.1. Composite Nanoparticles (Nanospheres, Hollow-Shell Particles, Reporter Probes)								
PE(s) Used	Sensing Element	Modification Methods	Electrode/Substrate	Path Of Modification	Sensor Construction	Analyte	Analysis Method	Reference
PAH(+); PSS(−)	Streptavidin/ AgNP-tagged carbon nanosphere (CNSs)	LbL	Au; CNS	Reporter probe: LbL onto CNS: PAH(+)-PSS(−); then in solution of Ag^+^ and citrate; then into streptavidin.	CNS/LbL: PAH-PSS/ AgNPs/streptavidin	DNA	Linear sweep voltammetry (LSV)	[83]
PAA(+); PSS(−)	SPE	LbL	SPE	LbL: Hollow shall PAA(+)-PSS(−) capsules loaded with Au(III) followed by reduction to obtain AuNPs@PAA(+)-PSS(−); magnetic Fe_3_O_4_ covered with APTES and polystyrene-co-acrylic acid (PSA(−)) latex particles (Fe_3_O_4_@APTES(+)@PSA(−)); then attachment of alkaline phosphatase (AP) labeled anti-DIG onto Fe_3_O_4_@APTES(+)@PSA(−); bonding of AuNPs@PAA-PSS and AP labeled anti-DIG @Fe_3_O_4_@APTES@PSA via Au-S bond	AP labeled anti-DIG@ Fe_3_O_4_@APTES@PSA@ AuNPs@PAA-PSS; magnetic collection; dissolution and determination	DIG (DNA)	DPSAV	[84]
PAH(+): PEI(+); PAA(−); PSS(−)	AChE and HRP	LbL/drop-casting	SPCE	LbL on the PSS(−) (as template): PAH(+)-PAA(−) or PEI(+)-PAA(−); then immobilization of enzyme onto polyelectrolyte sphere by mixing it into enzyme solution; then drop-casting onto SPCE	SPCE/LbL onto PSS: PAA-PAA or PEI-PAA/HRP or AChE	Pesticide and H_2_O_2_	AMP	[85]
PAH(+); PSS(−)	CdTeQD	LbL	GPTMS	Reporter probe: LbL onto polystyrene-co-acrylic acid (PSA) particles incubated with PAH(+)-PSS(−); then adding of CdTeQD onto PSA@PAH(+)-PSS(−); then conjugation of streptavidin onto PSA@PAH(+)-PSS(−)@CdTeQD; then electrostatic adsorption; then covered with BSA	PSA/LbL: PAH-PSS/CdTeQD/streptavidin	Target DNA	SWV	[86]
PAA(+); PSS(−)	DNA probe	LbL/adsorption	SPCE	Reporter probe: LbL onto polystyrene-co-acrylic acid (PSA) particles with PAA(+)-PSS(−); then adding of colloid AuNPs; then conjugation of DNA probe onto AuNPs; then electrostatic adsorption	PSA/LbL: PSS-PAA/AuNP/DNA	Target DNA	DPASV	[87]
**2.4.2. LbL Procedure in Other Sensor Architectures**								
PAH(+); PSS(−)	Proteus mirabilis bacteria	LbL/drop-casting	Si/SiO_2_	LbL on AuNPs: PAH(+)-PSS(−); then drop-casting onto Si/SiO_2_; then drop-casting of bacteria; then cross-linking with GSH	Si/SiO_2_/AuNPs@LbL: PAH-PSS/bacteria	Urea	POT	[88]
M13 phage(−); PEI(+)	GOx	LbL	SPAu	LbL on the cellulose acetate polymeric membrane by dialysis: SWCNTs@M13(−)-GOx@PEI(+); then wet contact-transfer-print onto SPAu	SPAu/LbL: SWCNTs@M13-GOx@PEI	Glucose	AMP	[89]
CMP(−); CS(+)	Urease	LbL/electropolymerization	SPCE	electropolymerization of PANI(+) on GCE; then immobilization of urease; then LbL: CMP(−)-CS(+);	GCE/PANI/urease/LbL: CMP-CS	Urea	POT	[90]
PDDA(+); CNNC(−)	PEDOT	LbL/electropolymerization	GCE	LbL: electrodeposition of PDDA(+)-then immersion into CNNC(−); then electrochemical polymerization of EDOT	GCE/LbL: PDDA-CNNC/PEDOT	Nitrite	AMP	[91]

**Table 5:** Summary of electrochemical sensors based on sensing layers obtained by drop-casting or dip-coating methods in a simple architecture: entrapping a sensing material (or redox mediator) in a polyelectrolyte matrix.

3.1. Sensing/Mediating/Transducing Component is Entrapped with PE								
3.1.1. Redox Mediators (Complex Compounds)–PE Composite								
PE(s) Used	Sensing Element	Modification Methods	Electrode/Substrate	Path of Modification	Sensor Construction	Analyte	Analysis Method	Reference
PDMDAAC (+)	Fe(CN)_6_^3−/4−^	Dip-coating	ITO	Dip-coating of the solution of PDMDAAC(+)@Fe(CN)_6_^3−/4−^	ITO/PDMDAAC@Fe(CN)_6_^3−/4−^	Water in organic solvents	IMP	[92]
PAH(+); PSS(−); CS(+)	Fe(CN)_6_^3−/4−^	Drop-casting	GCE	Drop-casting of the suspension of prepared porous material: CaCO_3_@PSS(−)@PAH(+)@Fe(CN)_6_^3−/4−^@CS(+)	GCE/CaCO_3_@PSS@PAH@Fe(CN)_6_^3−/4−^@CS	Ascorbic acid	AMP	[93]
PSS(−); Nafion^®^(−)	Ferrocenium (Fe(C_10_H_10_^+^)	Drop-casting	GCE	ferrocenium ion entangled and extracted with Nafion^®^ or PSS(−); then drop-casting of the Fe(C_10_H_10_^+^)(+)@Nafion^®^(−) or Fe(C_10_H_10_^+^)(+)@PSS(−)	GCE/Fe(C_10_H_10_^+^)@Nafion^®^ or Fe(C_10_H_10_^+^)@PSS	Nitrite	CV	[94]
**3.1.2. Me or MeO NPs–PE Composite**								
PADA(+)	AgNP	Drop-casting	GCE	Mixing solutions of PADA(+), TPDT (as reducing agent) and Ag^+^ to obtain PADA(+)@AgNP@TPDT; then drop-casting onto GCE	GCE/PADA@Ag@TPDT	H_2_O_2_ and 4-nitroaniline	SWV and LSV	[95]
PAA(+) sodium dodecyl sulphate (SDS(−)) surfactant	AgNP	Drop-casting	SPGE	solution of the AgNP and SDS (surfactant) (e.g., AgNP@SDS(−)) and PAA(+); then drop-casting onto SPGE	SPGE/AgNP@SDS@PAA	Thiourea	SWV	[96]
PADA(+)	PADA@Au50 Ag50NCs	Drop-casting	GCE	Drop-casting of solution of the bi-metallic Au/Ag nanoclusters embedded in PDDA(+): PADA(+)@Au50Ag50NCs	GCE/PADA@Au50Ag50NCs	NO	AMP	[97]
**3.1.3. Carbon-Based NPs–PE Composite**								
CS(+)	MWCNTs	Drop-casting	Au	Drop-casting suspension of MWCNTs@CS(+) onto Au	Au/MWCNTs@CS	Humidity	resistivity	[98]
PEDOT-S(−) (sulphonated PEDOT)	PEDOT-S@ MWCNTs	Drop-casting	GCE	Drop-casting of the suspension of the PEDOT-S(−)@MWCNTs onto GCE	GCE/PEDOT-S@MWCNTs	Nitrobenzenes	DPV;SWV	[99]
PHEMA-b-PDMAEMA(PHD(+))	MWCNTs@ PHD	Drop-casting	GCE	Drop-casting of the suspension of MWCNTs@PHD(+) onto GCE	GCE/MWCNTs@PHD	Bisphenol-A	AMP	[100]
PDDA(+)	Carbon nano-onions (CNO)	Drop-casting	GCE	Drop-casting of the suspension of CNO@PDDA(+) onto GCE	GCE/CNO@PDDA	Dopamine	DPV;SWV	[101]
Nafion^®^ (−)	MWCNTs@ Nafion^®^	Drop-casting	GCE	drop-casting of MWCNTs(^−^)@ Nafion^®^(−) onto GCE	GCE/MWCNTs@Nafion^®^	Pb, Cd	DPASV	[102]
PDDA(+)	rGO@PDDA	Drop-casting	GCE	Drop-casting of the suspension of rGO@PDDA(+) onto GCE	GCE/MWCNTs@PDDA	4-Nitrophenol	LSV	[103]
PDDA(+); PSS(−)	GO@PDDA or rGO@PSS	Dip-coating	Au	Dip-coating of the rGO@PDDA(+) or rGO@PSS(−) onto Au	Au/rGO@PDDA or rGO@PSS	Humidity	IMP	[104]
**3.1.4 Other–PE Composite**								
Cellulose sulphate (−); sodium alginate (−); poly(methylene-co-guanidine (+)	Cell entrapped in PEC membrane	Attach	Oxygen electrode	Suspension of biomass into solution of PEC; obtained gel placed onto oxygen electrode	Oxygen electrode/cell@PEC	Biotransformation based on Baeyer–Villiger oxidation-CBCH	AMP	[105]

**Table 6:** Summary of electrochemical sensors based on a polyelectrolyte-based sensing layer composed of more than one constituent and obtained by the drop-casting or dip-coating method.

3.2 Sensing Film Consisting of Three or More Components								
3.2.1. Sensing Film Comprising Me or MeO NPs								
PE(s) Used	Sensing Element	Modification Methods	Electrode/Substrate	Path of Modification	Sensor Construction	Analyte	Analysis Method	Reference
PDDA(+)	rGO@PDDA@ PtNPs	Drop-casting	GCE	Drop-casting of the rGO *@PDDA(+)@PtNPs onto GCE * rGO—graphene sheets	GCE/Gr@PDDA@Pt	Gallic acid	DPV	[106]
PSS(−)	rGO@PSS@ PdNP	Drop-casting	GCE	Drop-casting of the suspension of graphene rGO *@PSS(−)@PdNP * rGO—graphene	GCE/Gr@PSS@PdNP	Amaranth	DPV	[107]
PEI(+)	MWCNTs@ PEI@AuNP	Drop-casting	Au	Drop-casting of MWCNTs(−)@PEI(+)@AuNP	Au/MWCNTs@PEI@AuNP	Dopamine	DPV	[108]
PDDA(+)	rGO@PDDA@	Drop-casting	GCE	Reduction of the GO with PDDA(+); then addition of Au(III) in the rGO solution and reduction of Au(III) with PDDA(+); then drop-casting of the suspension of rGO@PDDA(+)@AuNP onto GCE	GCE/rGO@PDDA@AuNP	Aminophenol isomers	DPV	[109]
PDDMAC (+); Nafion^®^	Carbon ink@ Nafion^®^@AuNPs	Drop-casting	GCE	Electrodeposition of AuNPs from AuCl_4_^−^ and PDDMAC(+); drop-casting of the carbon ink@Nafion^®^(−)@AuNPs	GCE/carbon ink@ Nafion^®^@AuNPs	Dopamine	CV	[110]
**3.2.2. Sensing Film Comprising DNA**								
PAA(+); PSS(−)	DNA	Electropolym./Drop-casting	GCE	Electropolymerization of Neutral Red (NR) or PANI onto GCE; then drop casting of TC@PAA(+) or PSS(^−^)@DNA(^−^)	GCE/NR or PANI/TC@PAA or PSS@DNA	Doxorubicin	IMP	[111]
**3.2.3 Sensing Film Comprising an Enzyme**								
PAA(+); PEI(+); PAM(−)	GOx	Drop-casting	GCE	Entrapping of the ferrocene (Fc) with PE; then preparation of hybrid composite GO@MWCNTs@PE@Fc@GOx; then drop-casting of the GO@MWCNTs@PE@Fc@GOx	GCE/GO@MWCNTs@PE@Fc@GOx	Glucose	AMP	[112]
CS(+); kappa-carrageenan (−)	GOx	Drop-casting	Au	GOx encapsuled in PEC (CS(+)@kappa-carrageenan(−)); then mixed with AuNPs to obtain AuNPs@PEC@GOx; then drop-casting of the AuNPs@PEC@GOx onto Au	GCE/Au/AuNPs@PEC@GOx	Glucose	SWV	[113]
PDDA(+)	microperoxidase-11 (MP-11)	Drop-casting	GCE	Graphene@PDDA(+) mixed with AuNPs@DMPG 1,2-Dimyristoyl-sn-gly-cero-3-phosphatidil glycerol (DMPG) to obtain graphen@PDDA(+)@AuNPs@DMPG; then mixed with MP-11; then drop-casting onto GCE	GCE/graphen@PDDA/AuNPs@DMPG@MP-11	H_2_O_2_	CV	[114]
PAH(+)	GOx	Drop-casting	planar interdigitated electrode (IDE)	nanoparticles were coated with an initial layer of PAH; then dispersion of the PAH(+)@NPs into GOx; then Drop-casting of NPs@PAH(+)@GOx onto IDEs; then cross-linking in the vapor of GTH	IDE/NPs@PAH@GOx	Glucose	conductometry	[115]
SPAnNa(−)	HRP	Drop-casting	Au	poly[aniline-co-sodium N-(1-one-butyric acid) aniline]-zinc oxide (SPAnNa@ZnO) mixed with HRP; then drop-cast onto Au	Au/SPAnNa@ZnO@HRP	H_2_O_2_	AMP	[116]
Conjugated PEs: P3TOPS(−); aPPE(−);	GOx	Drop-casting	GCE	Preparation of complex (by mixing) of the SWCNTs, GOx and P3TOPS (−) or PPE(−); then drop-casting onto GCE	GCE/SWCNTs@PE@GOx	Glucose	AMP	[117]
PDDA(+)	AChE	Drop-casting	SPCE	GO and AuCl_4_^−^ chemically reduced with NaBH_4_ in the presence of PDDA(+); then AuNPs@PDDA@rGO mixed with AChE; then drop-cast onto SPCE	SPCE/AuNPs@PDDA@rGO@AChE	Paraoxone	AMP	[118]
PAH(+)	GOx	Drop-casting	ITO	SiO_2_NFs coated with PAH(+); then immersion into AuCl_4_^−^ followed by reduction by NaBH_4_; then mixing with GOx, L-cysteine and GTH; then immobilization onto ITO	ITO/SiO_2_@PAH@AuNPs@L-cysteine@GOx	Glucose	CV	[119]
PHEMA-b-PDMAEMA (PHD(+))	hemoglobin	Drop-casting	GCE	Drop-casting of the Hb@PHD(+)@MWCNTs GCE	GCE/Hb@PHD@MWCNTs	H_2_O_2_	AMP	[120]

* stands for all materials produced by same procedure, that are termed in same manner.

**Table 7:** Summary of electrochemical sensors based on the scaffolding polyelectrolyte-based layer, obtained by drop-casting or dip-coating methods.

3.3. PE in Preparation of Scaffolding Layer by Drop-Casting								
3.3.1. Redox Mediators (Complex Compounds)–PE Composite								
PE(s) Used	Sensing Element	Modification Methods	Electrode/Substrate	Path of Modification	Sensor Construction	Analyte	Analysis Method	Reference
PAA(+); dodecyl sulphate (DS(−)) as surfactant	PB	Drop-casting/electrodeposition	Graphite; ITO	Drop-casting of the PAA(+)@DS(−) onto electrode; then electrodeposition of the PB	Graphite or ITO/PAA@DS/PB	H_2_O_2_	AMP	[121]
PAA(+) derivatized with pyridine Os complex (PAO(+)); SDS (−) as surfactants	GOx	Drop-casting	Graphite	Precipitation of PAO(+) and SDS(−); then solution of PAO(+)@SDS(−) in dimethylformamide DMF onto G electrode; then drop-casting of GOx	Graphite/PAO@SDS/GOx	Glucose	CV	[122]
**3.3.2. Me or MeO NPs–PE Composite**								
silsesquioxane polymer SiPy(+)Cl(−)	HRP	Drop-casting	GCE	Drop-casting of the suspension of AuNPs stabilized with SiPy(+) onto GCE; then TLA (thiolactic acid); then immobilization of HRP via carbodiimide reaction (EDC/NHS)	GCE/AuNPs@SiPy/TLA/HRP	Catechol	DPV	[123]
**3.3.3. Carbon-Based Nanomaterials–PE Composite**								
Nafion^®^(−)	MWCNTs @ M(II)Ph; M = Zn, Co, Ni	Drop-coating/dip-coating	GCE	drop-coating of the solution of Nafion^®^(−) onto GCE; than in Nafion^®^(−)@MWCNTs; then in suspension of M(II)Ph(+)@Nafion^®^(−)	GCE/Nafion^®^@MWCNTs/M(II)Ph@Nafion^®^	H_2_O_2_	AMP	[124]
**3.3.4. Me or MeO NPs–Carbon-Based Nanomaterials–PE Composite**								
PDDA(+); PEI(+)	GOx and LOD	Drop-casting	Dual GCE	Drop-casting of the dispersion of the Pt@PDDA(+)@carbon mesoporous material (CMM) onto GCE; then drop-casting of the GOx and LOD@PEI(+); then cross-linking with GTH	GCE/Pt@PDDA@CMM/GOx or LOD@PEI	Glucose and L-lactate	AMP	[125]
PDDA (+)	Aptamer	Drop-casting	GCE	Drop-casting of the suspension of rGO@PDDA(+)@AgNP; then drop of solution of aptamer	GCE/rGO@PDDA@AgNP/aptamer	Chloramphenicol	LSV	[126]
PDDA(+)	HRP	Drop-casting	GCE	Graphene modified with PDDA(+); then electrostatic interaction with PtCl_6_^2-^; then PtCl_6_^2-^ microwave reduction; then drop-casting of the composite rGO@PDDA@PtNPs onto GCE; then drop-casting of the HRP	GCE/rGO@PDDA@PtNPs/HRP	H_2_O_2_	CV	[127]
PDDA(+)	rGO@PDDA(+)@PtNP	Drop-casting	SiO_2_/Ti or Au film	drop-casting of the rGO@PDDA(+) onto substrate; then immersion in the PtCl_6_^−^ followed by reduction of adsorbed Pt(IV) into PtNP by ascorbic acid	SiO_2_/Ti or Au film/rGO@PDDA@PtNP	Ascorbic acid (AA) dopamine (DA) and uric acid (UA)	AMP	[128]
CS(+)	Ab1	Drop-casting	GCE	drop-casting of the graphene@CS(+) onto GCE; then immersion of electrode in AuCl_4_^−^ followed by electroreduction; then drop-casting of Ab1 (anti-body)	GCE/graphene@CS@AuNPs/Ab1	Prolactin	DPV	[129]
PDDA(+)	Aptamer	Drop-casting	GCE	GO@PDDA(+) into AuCl_4_^−^ followed by chemical reduction to obtain GO@PDDA(+)@AuNPs; then drop-casting onto GCE; then incubation in aptamer solution	GCE/GO@PDDA@AuNPs/ssDNA	cDNA	SWV	[130]
PDDA(+); CS(+)	dsDNA	Dip-coating	PGE (pencil graphite electrode)	Various modification paths. Example: The PGEs were immersed into a suspension containing MWCNTs, TiO_2_NPs dispersed in PDDA(+); the electrodes were dipped into the dsDNA	PGE/MWCNTs@TiO_2_NPs@PDDA/dsDNA	Guanine and adenine	DPV	[131]
**3.3.5. PE or PEC as a Scaffolding Layer**								
PLL(+); Indigo tetra sulphonate (ITS)(−)	PLL(+)@GTH/ITS (−)	Drop-casting/electrodeposition	GCE	Drop-casting of the solution PLL(+)@GTH (as cross-linker) onto GCE; then electrodeposition of ITS(−)	GCE/PLL@GTH/ITS	Oxygen	RDE voltammetry	[132]
PSS(−); PEI(+)	Acridine orange (AO)	Dip-coating	GCE	Dip-coating of PEI(+); then dip-coating of PSS(−); then dip-coating AO(+)	GCE/PEI/PSS/AO	CTAB(−)	AMP	[133]
PAA(+)	CMWCNTs-AMWCNTs bonded via carbodiimide ester bond	Dip-coating/LbL	GCE	Dip-coating of GCE into PAA(+); then LbL: CMWCNTs-AMWCNTs/then EDC/NHS to form carbodiimide ester NOTE: AMWCNTs: MWNTs functionalized with amino terminated silanes (APS) CMWCNT: carboxylated MWCNT	GCE/PAA/LbL: CMWCNTs-AMWCNTs	NADH	AMP	[134]
**3.3.6 Other Uses of PE in Casting/Coating Mode**								
Nafion^®^(−); PAH(+)	Bienzymesensor: FFMNSs@PAH@MP11/GOx	Drop-casting	GCE	FF-MNSs modified with PAH(+) and MP11 to obtain FFMNSs@PAH(+)@MP11; then drop-casting onto GCE; then drop-casting GOx/then Nafion^®^(−);	GCE/FFMNSs@PAH@MP11/GOx/Nafion^®^	Glucose	AMP	[135]

**Table 8:** Summary of electrochemical sensors based on the application of polyelectrolytes or polyelectrolyte complexes as sensing layers.

3.4. PE or PEC as a Sensing Layer								
PE(s) Used	Sensing Element	Modification Methods	Electrode/Substrate	Path of Modification	Sensor Construction	Analyte	Analysis Method	Reference
CS(+); Pectin(−) (PC)	CS@PC	Drop-casting	GCE	Dispersion of biopolymer polyelectrolyte complex (CS(+)@PC(−))	GCE/CS@PC	Metronidazole and metribuzin	DPV	[136]
CS(+); Sulphonated CS(−) (SCS)	Cyclodextrin (CD)	Drop-casting	GCE	Homogenous solution of the of CS(+)@SCS(−)@CD onto GCE	GCE/CS@SCS and GCE/CS@SCS@CD	Atenolol	DPV	[137]
PDADMAC (+); CS-Cellulose sulphate(−)	PDMDAAC@CS	Sequential drop-casting	GCE	Sequential drop-casting of the PDADMAC(+) solution and CS(−) solution onto GCE	GCE/PDADMAC@CS	Nitrite	DPV	[138]
PAA(+); surfactant SDS(−)	SDS@PAA	Drop-casting	SPCE	Drop-casting of the suspension of PAA(+) and SDS(−)	SPCE/SDS@PAA	Xanthate	IMP	[139]
PAH(+)	PAH	Drop-casting	SPCE	Drop-casting of PAH(+) on oxidized SPCE	SPCE/PAH	NADH	DPV	[140]
PDDA(+)	PDDA	from the solution	GCE	PDDA(+) in sample solution	GCE covered with PDDA during analysis	uric acid and ascorbic acid	DPV	[141]
PAA(+); HPA (+)	Hydrazinium poly acrylate (HPA)	Drop-casting	SPCE	Drop-casting of the PAA(+) or HPA(+) onto SPCE	SPCE/PAA or HPA	Form aldehyde	CV	[142]
PBI-BA(−)	poly[N-(1-one-butyric acid) benzimidazole] (PBI-BA)	Drop-casting	Au	Drop-casting of the solution of the PBI-BA(−) or graphene modified PBI-BA(−) (PBI-BA(−)@ graphene) onto Au	Au/PBI-BA or graphene@PBI-BA	H_2_O_2_	AMP	[143]
PAH(+)	ssDNA	Drop-casting	Chip (pSi-SiO_2_)	Drop-coating of the PAH(+) onto chip/then immobilization of ssDNA(^−^)	Chip/PAH/ssDNA	cDNA	IMP	[144]

**Table 9:** Summary of electrochemical sensors based on other methods that involve polyelectrolytes in sensor architectures.

3.5. Other Method Involved in Application of PEs in Sensor Architectures								
3.5.1. Electrospinning and Spin-Coating of PE onto a Working Electrode								
PE(s) Used	Sensing Element	Modification Methods	Electrode/Substrate	Path of Modification	Sensor Construction	Analyte	Analysis Method	Reference
Crosslinked and quaternized QC-P4VP(+)	AgNPs	Electro spinning/dip-coating	Au	Electrospinning of P4PVP; then crosslinking and quaternization with 1,4-dibromobutane (DBB) to obtain QC-P4VP(+); then dip-coating into Ag^+^ followed by reduction by UV lamp	Au/QC-P4VP@AgNPs	Humidity	IMP	[145]
PAH(+)	PA6@PAH(+)@MWCNTs	Electrospinning	ITO	Electrospinning of polyamide (PA6) and PAH(+) onto ITO; then immersion into dispersion of the MWCNTs	ITO/PA6@PAH@MWCNTs	Dopamine	DPV	[146]
PAA(+) SDS(−) surfactant	HRP	Spin-coating	Si coated with Pd, Ti, Au	spin-coated redox-active glycoppolyelectrolyte containing Os(bpy)_2_ (GOsPA) mixed with SDS(−) onto electrode; then building of the protein building blocks such as Con A, Os-Con A; then immobilization of HRP	Au/GOsPA-SDS/Con A or Os-Con A/HRP	H_2_O_2_	CV; AMP	[147]
PDDA(+); PVS(−)	Silica@PEC	Spin coating/drop-casting	GCE; glass	mixing solution of tetraethyl orthosilicate (TEOS) and PDDA(+) or PEC (PDDA(+)@PVS(−)) during preparation of silica sol (in various ratio depending on ion-exchange power); then addition of adenine; then spin-coating onto electrode; then removing adenine from film in ethanol.	GCE/silica@PEC	Adenine and guanine	DPV;SWV	[148]
**3.5.2. PEs in Construction of Reporter Probes, Nanoprobes, etc.**								
PDDA(+)	DNAzyme/PtNPs/CNTs	Hybridization	Au	Capture probe (electrode): hybridization of target DNA onto Au electrode modified with capture DNA probe Reporter probe: mixture of CNTs(−) and PDDA(+) to obtain CNTs(−)@PDDA(+); then mixing with PtNPs; then immobilization of DNAzyme (DNA labeled with enzyme) by mixing; then immobilization onto modified Au electrode by hybridization.	Reporter probe (nanoprobe): CNTs@PDDA@PtNPs@DNAzyme	DNA	AMP	[149]
PDDA(+)	Anti-HigG	Drop-casting	SPCE	Capture probe (electrode): drop-casting of the Au(III) onto SPCE followed by electroreduction; then immobilization of the anti-HigG; then BSA (blocking surface); Reporter probe (nanoprobe): Prussian blue (PB) functionalized mesoporous carbon nanosphere (MCN) (obtained using silica template) (MCN@PB(−)); then coated with PDDA(+); then reduction of the AuCl_4_^−^ with citrate to obtain MCN@PB(−)@AuNPs; then anti-HigG and GOx through the inherent interaction between Au NPs and protein	Reporter probe (nanoprobe): MCN@PB/PDDA@AuNPs@HigG and GOx	HigG	DPV	[150]
PDDA(+); PSS(−)	FC@PE@Ab2	Drop-casting	GCE	Capture probe (electrode): immobilization of Ab1 by amidation onto oxidized GCE(−) Reporter probe (nanoprobe): CaCO_3_ (as templates) nanoparticles mixed with PDDA(+), PSS(−) and FC; then mixing of the obtained modified CaCO_3_@FC@PDDA(+)@PSS(−) with Ab2	Reporter probe (nanoprobe): CaCO_3_@FC@PDDA@PSS@Ab2	Interleukin-6 (IL-6)	SWV	[151]
PAA(+)	Gene sensing material		Rubber	PEDOT embedded electrospun rubber; then grafting PAA(+) as brushes; then potentiodynamically polymerized ssDNA(^−^)	Rubber/PEDOT/PAA/gene sensing material	Non-Hodgkin lymphoma gene (cDNA)	IMP	[152]

## Abbreviations

**PEs:**	
MP	carboxymethylpullulan;
CNCC	carboxylated nanocrystalline cellulose;
CS	chitosan;
HA	humic acid;
PAA(+)	poly(acrylamide);
PAA(−)	poly(acrylic acid);
PADA	poly[acrylamide-co-(diallyldimethylammonium chloride)];
PAH	poly(allylamine hydrochloride);
PAM	polyacrylamide;
PAMAM	poly(amidoamine) dendrimer;
PAS	sodium poly(anethol sulphonate);
PC	pectin;
PDADMAC	poly(diallyldimethylammonium chloride);
PDDA	poly(diallyldimethylammonium chloride);
PEI PHD	poly(ethylenimine); poly(hydroxyethyl methacrylate-poly[2-(dimethylamino)ethyl methacrylate;
PHEMA-b PDMAEMA PSS	poly(hydroxyethyl methacrylate; poly[2-(dimethylamino)ethyl methacrylate;poly(styrene sulphonate);
PQ11	poly [(2-ethyldimethylammonioethyl methacrylate ethyl sulphate)-co-(1-vinylpyrrolidone);
PVP	poly(vinyl pyrrolidone);
PVS	poly(vinylsulphate);
SCS	sulphonated chitosan;
SiPyCl	3-*n*-propylpyridinium chloride silsesquioxane.
**Carbon Nanomaterials:**	
CNS	carbon nanosphere;
CNO	carbon nano-onions;
ErGO	electrochemically reduced graphene oxide;
GO	graphene oxide;
MWCNTs	multi-wall carbon nanotubes;
rGO	reduced graphene oxide;
SWCNTs	single-wall carbon nanotubes.
**DNA:**	
cDNA	complementary DNA;
ssDNA	single stranded DNA.
**Enzymes:**	
AChE	acetylcholinesterase;
ChOx	choline oxidase;
HRP	horse radish peroxidase;
GOx	glucose oxidase;
LOx	lactate oxidase;
SOD	sarcosine oxidase.
**Electrode:**	
FET	field-effect transistor;
FTO	fluorine doped tin oxide;
GCE	glassy carbon electrode;
IDE	planar interdigitated electrode;
ITO	indium tin oxide;
SPAu	screen-printed gold electrode;
SPCE	screen-printed carbon electrode;
SPGE	screen-printed graphite electrode.
**Other:**	
APTES	3-aminopropyl-triethoxysilane;
BSA	bovine serum albumin;
CD	cyclodextrin;
GTH	glutaraldehyde;
MB	methylene blue;
MG	methylene green;
MPA	mercaptopropionic acid;
PANI	polyaniline;
PB	Prussian blue;
PEDOT	poly(3,4-ethylenedioxythiophene);
PSA	polystyrene-co-acrylic acid;
SDS	sodium dodecyl sulphate;
S-PEDOT	sulphonated poly(3,4-ethylenedioxythiophene);
TEOS	tetraethyl orthosilicate.
**Electroanalytical Methods:**	
AMP	amperometry;
CV	cyclic voltammetry;
DPASV	differential pulse anodic stripping voltammetry;
DPV	differential pulse voltammetry;
EIS	use of the electrochemical impedance spectroscopy;
IMP	impedimetry;
LSV	linear sweep voltammetry;
POT	potentiometry;
SWV	square-wave voltammetry
